# Zinc-finger domains of the transcriptional repressor KLF15 bind multiple sites in rhodopsin and IRBP promoters including the CRS-1 and G-rich repressor elements

**DOI:** 10.1186/1471-2199-6-15

**Published:** 2005-06-17

**Authors:** Deborah C Otteson, Hong Lai, Yuhui Liu, Donald J Zack

**Affiliations:** 1 Guerrieri Center for Genetic Engineering and Molecular Ophthalmology at the Wilmer Eye Institute, Johns Hopkins University School of Medicine; 600 North Wolfe Street; Baltimore, MD 21287 USA; 2 Department of Ophthalmology, Johns Hopkins University School of Medicine; 600 North Wolfe Street; Baltimore, MD 21287 USA; 3 Departments of Neuroscience, and Molecular Biology and Genetics; Johns Hopkins University School of Medicine; 600 North Wolfe Street; Baltimore, MD 21287 USA; 4 College of Optometry, University of Houston; Houston, TX 77204 USA; 5 Department of Genetics, Stanford University School of Medicine; Stanford, CA 94305 USA

## Abstract

**Background:**

In the retina, many of the genes that encode components of the visual transduction cascade and retinoid recycling are exclusively expressed in photoreceptor cells and show highly stereotyped temporal and spatial expression patterns. Multiple transcriptional activators of photoreceptor-specific genes have been identified, but little is known about negative regulation of gene expression in the retina. We recently identified KLF15, a member of the Sp/*Krüppel*-like Factor family of zinc-finger containing transcription factors, as an *in vitro *repressor of the promoters of the photoreceptor-specific genes *rhodopsin *and *IRBP/Rbp3*. To gain further insight into the mechanism of KLF15-mediated regulation of gene expression, we have characterized the binding characteristics and specificity of KLF15's DNA binding domains and defined the KLF15 binding sites in the *rhodopsin *and *IRBP *promoters.

**Results:**

In EMSA and DNAseI footprinting assays, a KLF15-GST fusion protein containing the C-terminal zinc-finger domains (123 amino acids) showed zinc-dependent and sequence-specific binding to a 9 bp consensus sequence containing a core CG/TCCCC. Both the bovine *rhodopsin *and *IRBP *promoters contained multiple KLF15 binding sites that included the previously identified CRS-1 and G-rich repressor elements. KLF15 binding sites were highly conserved between the bovine, human, chimp and dog *rhodopsin *promoters, but less conserved in rodents. KLF15 reduced luciferase expression by bRho130-luc (containing 4 KLF15 sites) and repressed promoter activation by CRX (cone rod homeobox) and/or NRL (neural retina leucine zipper), although the magnitude of the reduction was smaller than previously reported for a longer bRho225-luc (containing 6 KFL15 sites).

**Conclusion:**

KLF15 binds to multiple 9 bp consensus sites in the *Rhodospin *and *IRBP *promoters including the CRS-1 and G-rich repressor elements. Based on the known expression pattern of KLF15 in non-photoreceptor cells, we hypothesize an *in vivo *role for KLF15 in repressing photoreceptor-specific gene expression in the inner retina.

## Background

Photoreceptors are the highly specialized sensory receptors of the retina and express a unique array of genes that enable them to convert light energy into a neural signal. Many of these genes, including those encoding components of the phototransduction cascade [e.g. rhodopsin (Rho), transducin, arrestin, α– and β-phosphodiesterase (PDE)] and those involved in retinoid recycling [e.g. interphotoreceptor retinoid binding protein (IRBP/RBP3)], are expressed only in photoreceptor cells in the retina and in a subset of cells in the pineal gland [1-5]. In addition, these genes have highly stereotyped temporal and spatial patterns of expression during retinal development [6-8] that are transcriptionally regulated [7,9,10]. We are interested in understanding the transcriptional networks that regulate photoreceptor-specific gene expression, not only as a model for cell-specific gene regulation, but to gain insight into the mechanisms that regulate photoreceptor differentiation and neuro-degenerative retinal disease.

The proximal promoter regions of both *Rho *(-225 to +70 base pairs relative to the transcription start site) [11-13] and *IRBP *(-123 to +18 bp) [6,14] are sufficient to drive photoreceptor-specific expression of reporter genes in transgenic mice. Using DNAse I protection assays, binding sites for both retina-enriched and more widely expressed proteins have been identified on these promoters [15-22] and include both positive and negative regulatory elements [17-19,23-29]. Among the positive regulatory elements, Ret-1, BAT-1 and PCE-1 each contain a core ATTA sequence that can be bound by homeodomain containing transcription factors including CRX [30], RX/RAX [31], ERX [32], QRX [33] and OTX2 [18,34]. Binding sites for the basic-leucine zipper transcription factor NRL have been identified in the promoters of *rhodopsin *[35,36] as well as other photoreceptor-specific genes including *arrestin *[26] and the rod-specific α- and β-*PDE *[37,38]. Combinatorial effects by CRX and NRL result in synergistic increases in *Rho *promoter activation *in vitro *that likely contribute to the high levels of expression in rod photoreceptors *in vivo *[29,30,39] Both *Crx *and *Nrl *play key roles in photoreceptor development and survival: in *Crx *knock-out mice, rod photoreceptors fail to elaborate outer segments and degenerate and the mice lack detectible visual function [40]; in *Nrl *knock-out mice, rod photoreceptors apparently fail to differentiate (although there is an increase in cone-like cells) and mice have severely reduced visual function [41]. In addition, mutations in both *CRX *and *NRL *have been identified in patients with a variety of inherited retinal degenerative diseases including autosomal dominant and recessive *retinitis pigmentosa*, Leber congenital *amaurosis *and cone-rod dystrophy [42-45].

Although transcriptional repression plays an important role in development and cell fate determination in the central nervous system and inappropriate de-repression of gene expression can contribute to disease [46,47], relatively little is known about negative regulation of photoreceptor-specific gene expression. Negative regulatory elements, including the CRS-1 site in the *rhodopsin *promoter [21] and the G-rich repressor in the *IRBP *promoter [20] are bound by nuclear proteins present in both retinal and non-retinal tissues, although the identities of the specific transcription factors have not been established. The zinc-finger transcription factor MOK2 binds to sites in the promoter and intron 1 of *IRBP *and represses transactivation of reporter constructs containing these elements *in vitro *[48]. Other proteins interact with CRX (e.g. ATXN-7 [49] and BAF [50]) or NRL (e.g. FIZ-1 [51]) and can reduce their ability to activate transcription, but these appear to repress via protein/protein interactions rather than binding directly to the promoter. We recently identified KLF15, a member of the Sp/KLF family of zinc-finger containing transcription factors, as a repressor of both the *rhodopsin *and *IRBP *promoters *in vitro *[52].

KLF15 is expressed in both retinal and non-retinal tissues and, like other members of the Sp/KLF family, is characterized by the presence of three *Krüppel*-type zinc-finger domains at the C-terminus [52-54]. Members of this gene family have been characterized as regulators of both tissue-specific and ubiquitous genes and can function as either transcriptional activators, repressors or both depending on promoter context [55,56] KLF15 appears to be bi-functional, as it represses the *Rho *and *IRBP *promoters [52] and the kidney-specific *Clc-K1 *and *Clc-K2 *promoters [53] but activates the *Glut4 *(glucose transporter 4) promoter [54]. KLF1/EKLF [57,58], KLF8 [59] and KLF15 [54] all bind a core CACCC site *in vitro*, although this is shorter than the 9 bp site predicted for a three zinc-finger containing protein and is likely to provide only limited specificity. To gain further insight into the mechanism of KLF15-mediated regulation of gene expression, we have characterized the binding characteristics and specificity of KLF15 and defined the KLF15 binding sites in the *Rho *and *IRBP *promoters.

## Results

### EMSA analysis of KLF15 binding

**Figure 1:**
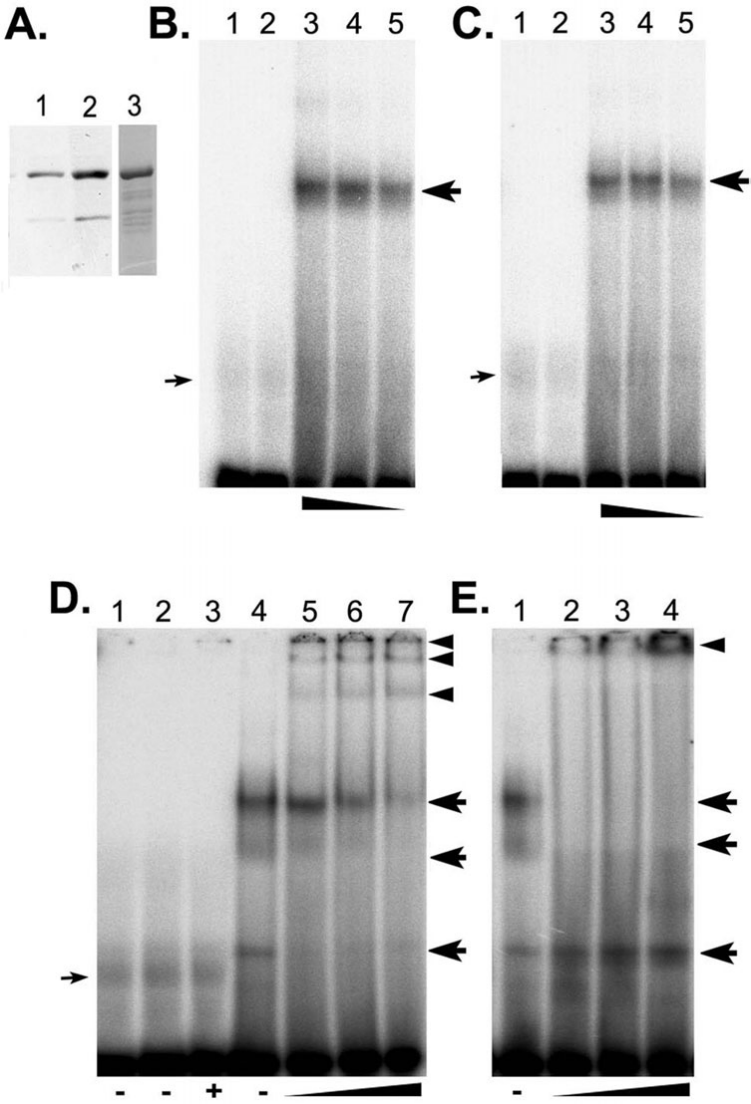
EMSA analysis of KLF15 binding. (A) KLF15-ZF fusion protein. Lanes 1 and 2 show western blots of crude bacterial lysates at 1.5 and 2 hours following induction of fusion protein expression. Anti-GST antibodies detect a major band of the anticipated size and additional lower molecular weight bands. Lane 3, Coomassie stained gel of affinity purified KLF15-ZF-GST fusion protein showing enrichment of major band containing full length fusion protein. (B) EMSA using ^32^P-labeled oligomers (bRho29) containing a 29 bp fragment from bovine rhodopsin promoter (-94 to -66). Lane 1: no protein; Lane 2: 67.5 ng GST; Lane 3–5: 100, 50, 25 ng KLF15-ZF-GST. (C) Same as (B) except ^32^P-labeled oligonucleotide (hRho29) contained corresponding sequence from human rhodopsin promoter. (D) Supershift using ^32^P-bRho29 oligonucleotide and anti-KLF15 antibodies. Lane 1, no protein; Lane 2, 33 ng GST protein; Lane 3, 33 ng GST + anti-KLF15; Lane 4, 50 ng KLF15-ZF-GST protein; Lanes 5–7, 50 ng KLF15-ZF-GST protein plus increasing amounts of anti-KLF15. (E) Supershift using ^32^P-bRho29 oligonucleotide and anti-GST antibodies. Lane 1, 50 ng KLF15-ZF-GST protein; Lanes 2–4, 50 ng KLF15-ZF-GST protein plus increasing amounts of anti-GST. Large arrows, KLF15 shifted bands; small arrows, non-specific bands; arrowheads, supershifted bands.

DNA binding properties of KLF15 were analyzed by electrophoretic mobility shift assays (EMSA) using GST-tagged, human KLF15 fusion protein. Constructs for the full-length KLF15 fusion protein expressed poorly in bacterial cells and yielded a highly degraded protein. Therefore, a recombinant fusion protein (KLF15-ZF) containing the C-terminal 123 amino acids including the three C-terminal zinc-finger domains fused to GST was expressed and purified from bacteria. Western blot of crude bacterial lysates using anti-GST antibodies showed minor degradation of the fusion protein prior to purification; however, the majority of the protein recovered following affinity purification consisted of intact KLF15-ZF fusion protein (Fig. 1A).

Comparison of the proximal promoter regions showed that the 29 bp fragment of the bovine *Rho *promoter (bRho29) used as bait in the yeast one-hybrid assay that identified KLF15 [52] shared high sequence identity with the equivalent element from the human *rhodopsin *promoter (hRho29), differing by only 5 bp. In EMSA, KLF15-ZF fusion protein shifted oligomers for both bRho29 (Fig. 1B) and hRho29 (Fig. 1C) and these could be supershifted by addition of antibodies against KLF15 (generous gift of S. Uchida [53]) (Fig. 1D) or GST (Fig. 1E). The lower molecular weight band that was supershifted by the anti-KLF15 but not by anti-GST antibodies most likely resulted from low levels of protein that had lost the N-terminal, GST-tag, but retained the C-terminus of the protein containing the DNA binding domain and the anti-KLF15 antibody recognition site.

### Zinc dependence of KLF15 binding

**Figure 2:**
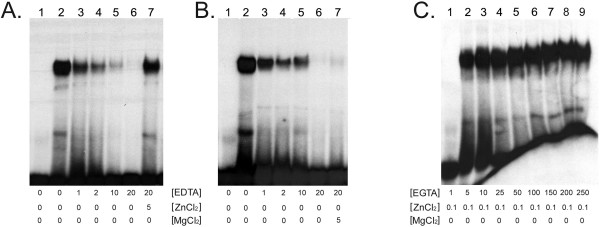
Effects of zinc chelation on KLF15 Binding. A. EMSA using ^32^P-labeled oligomers (bRho29) containing -96 to -66 bp fragment from bovine rhodopsin promoter. Lane 1, GST protein; Lane 2, KLF15-ZF-GST protein, Lanes 3 -7 same as 2 with EDTA and ZnCl_2 _(mM) as indicated. B. Same as A, but with MgCl_2_. C. Same as A, but with EGTA and ZnCl_2_. The altered migration of the unbound oligonucleotides is attributable to the salt effects of high concentrations of EGTA.

The secondary structure of zinc-finger domains and as well as their ability to bind DNA is dependent on the chelation of Zn^+2 ^ions by Cys2-His2 residues within each zinc finger [60,61]. To determine if DNA binding by KLF15-ZF was in fact Zn^+2 ^dependent, aliquots of the fusion protein were diluted in EDTA to chelate divalent cations and subsequently added to binding reactions containing ^32^P-labeled bRho29 or hRho29 oligonucleotides. Increasing concentrations of EDTA resulted in a dose-dependent reduction in KLF15-ZF binding to bRho29 (Fig. 2A, B) or to hRho29 (data not shown), with binding completely eliminated when protein was prepared in 50 mM EDTA, resulting in 20 mM EDTA in the final binding reaction. DNA binding was reconstituted by addition of 5 mM ZnCl_2 _(Fig. 2A) or ZnAcetate (data not shown), but not MgCl_2 _(Fig. 2B) to the binding reactions. As an additional test of cation specificity, we tested KLF15 binding activity in the presence of EGTA, a chelator that preferentially binds Ca^+2^. No loss of KLF15-ZF binding was observed in the presence of up to 250 mM EGTA (Fig. 2C). It is interesting to note that the retina and other ocular tissues contain unusually high levels of zinc compared to other tissues and zinc deficiency has been implicated with various retinopathies including macular degeneration, night blindness and *retinitis pigmentosa *[62,63]. Chelation of intracellular zinc can modulate conformation and DNA binding activity of the zinc-finger transcription factor p53 in cultured cells [64,65]; therefore, physiological zinc deficiencies could result in loss of DNA binding activity of zinc-finger proteins *in vivo*.

### Sequence specificity of KLF15 binding

**Figure 3:**
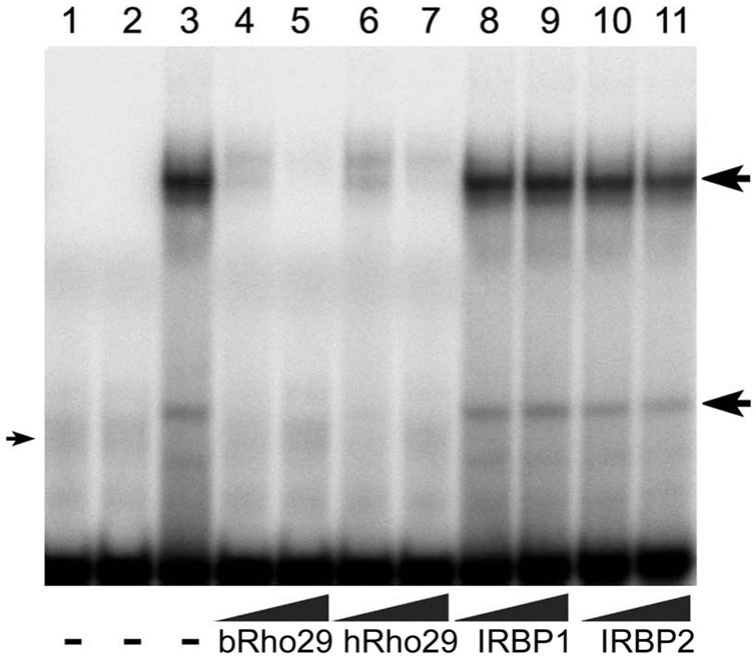
Competitive EMSA using (A) ^32^P- bRho29 oligonuclotides (bovine). Lane 1, no protein; lane 2, GST protein control; lane 3, KLF15-ZF-GST without competitor; lanes 4 -11, same as lane 3 but with unlabeled competitors (50 ×, 250 × fold-excess) as indicated. Large arrows indicate specific bands; small arrow indicates non-specific band present in negative (no protein and GST only) controls; IRBP1, MOK2 site from bovine IRBP promoter (5'-GGACAGGATTAAAGGCTTACTGGAG-3'); IRBP2, MOK2 site from bovine IRBP intron1 (5'-GGACTTGTCAGGGCCTTTA-3')

Sequence specificity of KLF15-ZF binding was examined by EMSA using a series of unlabeled oligomers as competitors (Fig. 3). The addition of 250-fold excess of cold bRho29 or hRho29 effectively competed with the radiolabeled bRho29 probe for KLF15 binding. We also tested oligomers IRBP1 and IRBP2 that contained binding sites for the zinc-finger transcription factor MOK2 and are located in the promoter and intron 2 of IRBP respectively [48]. Neither of these heterologous oligomers competed with radiolabeled bRho29 (Fig. 3) or hRho29 probes (data not shown).

**Figure 4:**
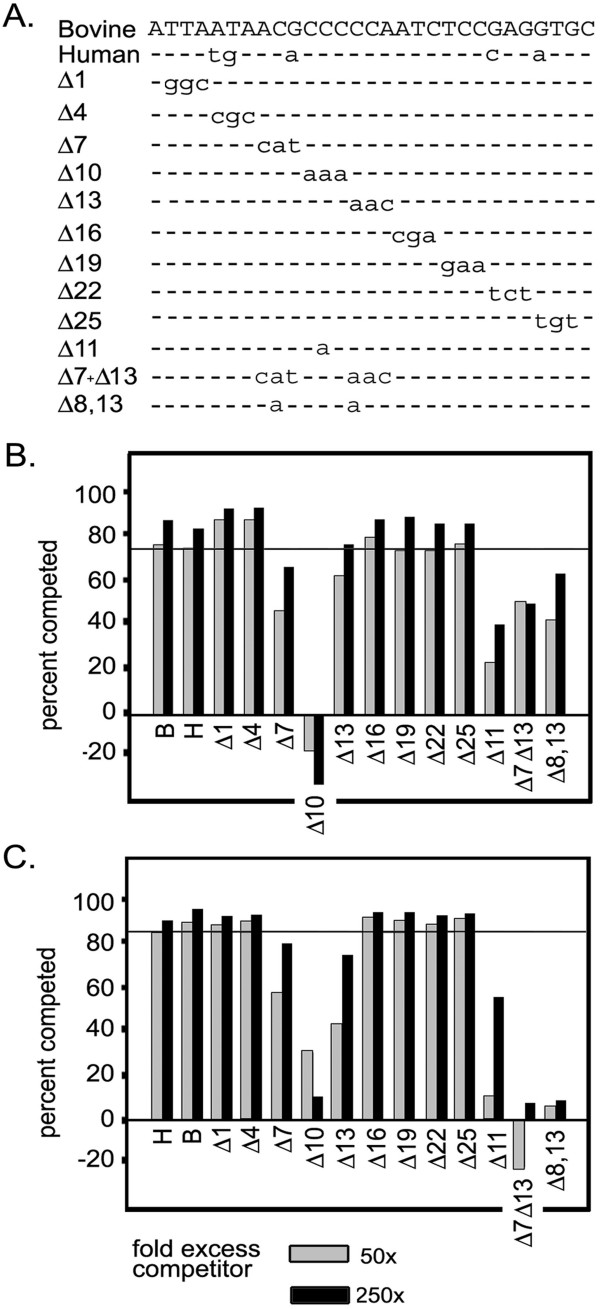
Sequence specificity of KLF15 binding: mutational analysis of the bovine *rhodopsin *promoter element (bRho29). (A) Sequence of oligomers used in EMSA analysis showing mutations analyzed in B and C. (B) Graph summarizing results of competitive EMSA using ^32^P-labeled bRho29 and unlabeled mutant oligomers as indicated. B, bRho29 competitor; H, hRho20 competitor. Bars show % of shifted oligonucleotide competed by addition of cold competitor. Bars with negative value reflect an increase in the amount of oligonucleotide shifted following the addition of cold competitor. (C) Same as B, except ^32^P-labeled hRho29 oligonucleotide was used.

To identify the specific bases within bRho29 and hRho29 that were critical for binding, we generated a series of scanning oligomers, each containing a triplet of mutated bases (Fig. 4A). Because an individual zinc-finger domain typically binds to a DNA triplet, this approach was selected to maximize the likelihood that the mutations would disrupt protein/DNA interactions. Oligomers with 1 or more mutated bases within the central C-rich sequence (bovine: ACG CCC CCA; human ACA CCC CCA) had reduced ability to compete with the wild-type bRho29 (Fig. 4B) or hRho29 (Fig. 4C), whereas oligomers with mutations of any other triplet competed as well as wild-type.

**Figure 5:**
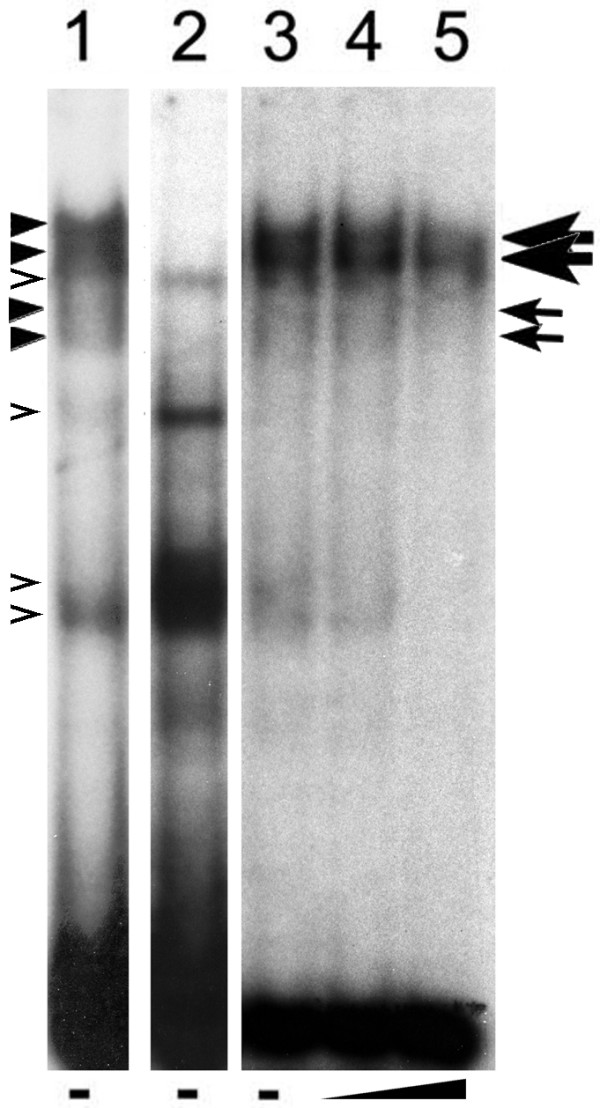
Binding of KLF15 site by retinal proteins in EMSA. All lanes contain equal amounts of total retinal extract from wild-type adult mouse retina. Lanes 1, 3, 4 and 5, ^32^P-labeled bRho29 oligo; lane 2, ^32^P-labeled Δ10 oligo containing mutated KLF15 binding site; cold Δ10 oligo as competitor was added to lane 4, at 20-fold excess and lane 5, at 200-fold excess. Open arrowheads, bands shifted with both wild-type and mutant oligos; solid arrowheads, bands observed only with wild-type oligos; large and small arrows respectively indicate major and minor bands not competed by addition of excess mutant oligo. Discontinuities in the image resulted from removal of irrelevant lanes.

To test if there was binding activity in retinal proteins that was specific to the KLF15 binding site, we performed EMSA using a total retinal extract with either the wild-type bRho29 oligo or the Δ10 oligo (see Fig. 4A) containing a mutated KLF15 binding site (Fig. 5). With both the wild-type and mutant oligos, there were multiple shifted bands observed, however mutation of the KLF15 binding site eliminated several of the major bands. Bands that were unique to the wild-type oligo were not abolished by the addition of up to 200-fold excess of the Δ10 oligo as a competitor, while those that were common to both were reduced in intensity to near background levels. This is consistent with our findings that Δ10 was unable to compete with the wild-type bRho29 for binding to the KLF15-ZF fusion protein in competitive EMSA (Fig. 4B). We observed that with the wild-type oligo, those bands that were dependent on the presence of an intact KLF15 binding site showed the greatest intensity; in contrast, when the KLF15 binding site was mutated, those bands that were not dependent on the presence of the KLF15 binding site were increased in intensity.

### KLF15 binding sites in rhodopsin and IRBP promoters

**Figure 6:**
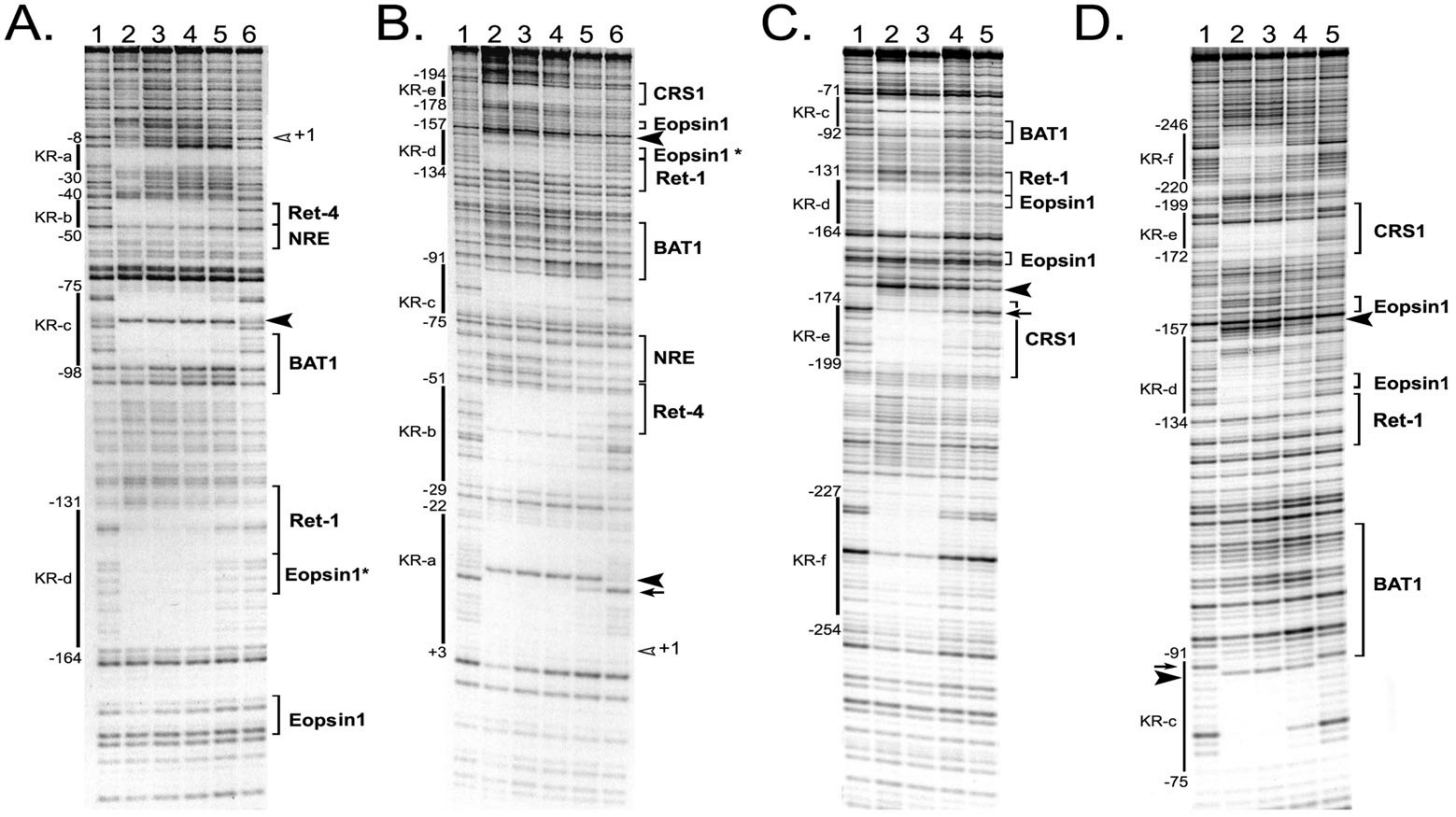
DNAse I footprint analysis of KLF15 binding sites in the *rhodopsin *promoter. (A) A PCR fragment spanning -225 to +70 bp of the bovine RPPR was end labeled with ^33^P at the -225 bp end (forward strand) and incubated with 250, 125, 62.5 and 31.25 ng of purified KLF15-ZF fusion protein (lanes 2–5). Lanes 1 and 6 contained no fusion protein. Protected regions are designated by lines on the left of the image with the nucleotide position of each protected region indicated. Previously identified protein binding sites are indicated by brackets. Solid arrowheads indicate novel hypersensitive sites resulting from KLF15-ZF binding; small arrows indicate hypersensitive sites that are lost or altered by KLF15-ZF binding. Transcription start site (+1) is indicated by an open arrowhead. (B) Same as (A) except the -225 to +70 fragment was labeled at the +70 end (reverse strand). (C) Same as (A) except the PCR fragment used spanned -315 to -31 and was end-labeled with ^33^P at the -315 end (forward strand) and lanes 1 and 5 contained no protein. (D) Same as (C) except the -315 to -31 fragment was labeled at the -31 end (reverse strand).

**Figure 7:**
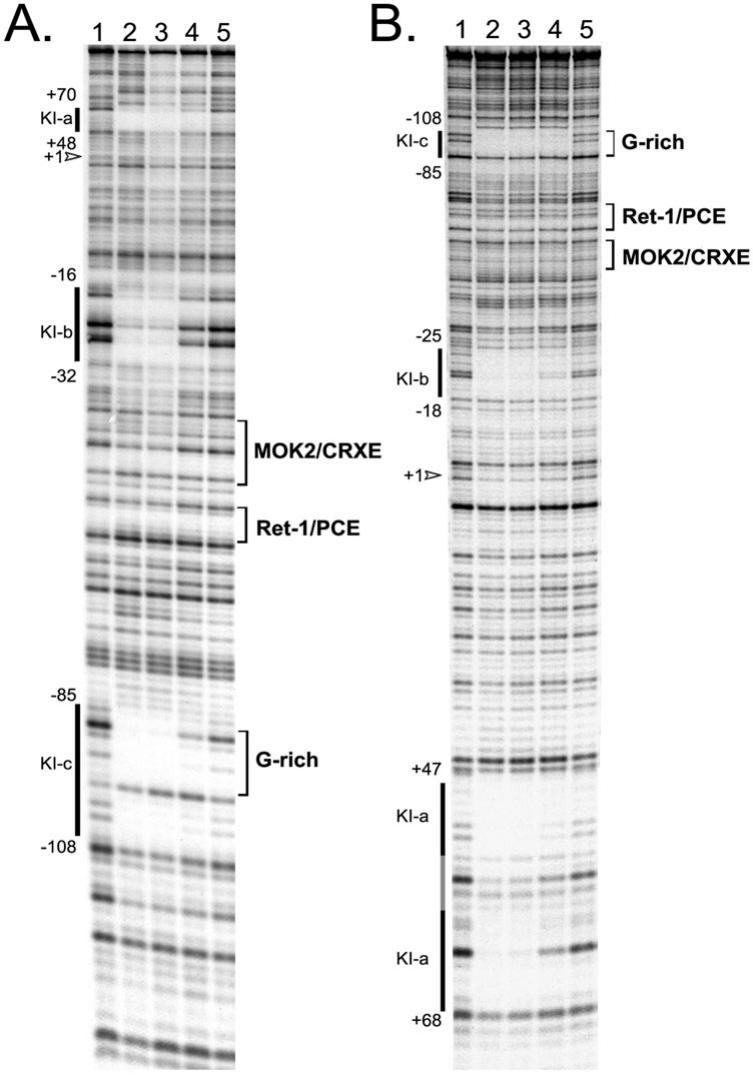
DNAse I footprint analysis of KLF15 binding sites in the *IRBP *promoter. (A) A PCR fragment spanning -132 to +70 bp of the bovine *IRBP *promoter was end labeled with ^33^P at the -132 bp end (forward strand) and incubated with 250, 125 and 62.5 ng of purified KLF15-ZF fusion protein (lanes 2–4). Lanes 1 and 5 contained no fusion protein. Protected regions are designated by bars on the left of each panel with the nucleotide position of each protected region indicated. Previously identified protein binding sites are indicated by brackets. Transcription start site (+1) is indicated by an open arrowhead. (B) Same as (A) except the fragment was end-labeled with ^33^P at the +70 end (reverse strand). The bands present in KI-a (indicated by grey bar in Panel A) were also present in negative control lanes containing undigested probe (data not shown).

DNAse I footprinting was used to identify KLF15 binding sites in the bovine *Rho *and *IRBP *promoters. Using two overlapping fragments of the bovine RPPR (-225 to + 70 bp and -315 to -31 bp) as templates, KLF15-ZF protected six distinct sites (designated KR-a to KR-f) that were generally similar in their boundaries on both the forward and reverse strands (Fig. 6 A–D). In the *IRBP *proximal promoter, KLF15 protected three sites (KI-a, KI-b, KI-c) on both forward and reverse strands (Fig. 7A, B). Examination of the sequences protected by KLF15-ZF showed that all contained extended C-rich or G-rich sequences (Fig. 10A), consistent with the site identified by competitive EMSA. To identify a consensus binding site, we analyzed the sequences of the C-rich strands of the nine KLF15 protected regions using Target Explorer software [66]. Assuming a 60% AT, 40% GC content in the genome and a 9 bp binding site, we identified the top 4 matrices (Additional file: 1). All of the matrices included the site identified by competitive EMSA and, when used to evaluate the oligos tested in competitive EMSA, could discriminate between the strong competitors and weak/non-competitors.

**Figure 8:**
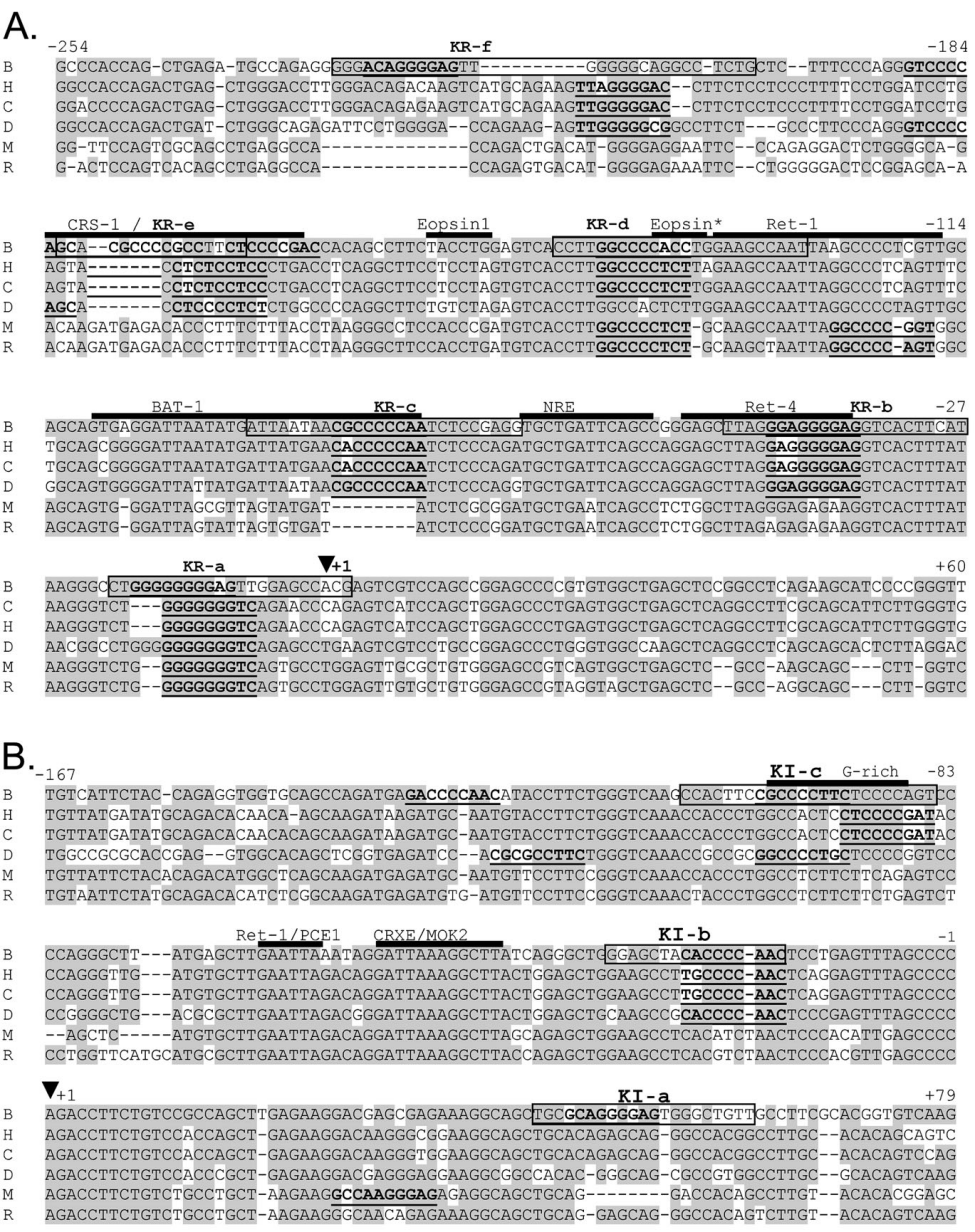
Alignment of (A) *rhodopsin *and (B) IRBP proximal promoters. KLF15 protected sites in bovine promoter are boxed; 9 bp KLF15 binding sites predicted using Target Explorer Matrix with a cutoff score of 4.86 (Table 1; see also Additional file: 1) are underlined and in bold; at locations where multiple overlapping binding sites were predicted, only the one with the highest score is marked. Previously identified protein binding sites/regulatory elements are indicated by heavy solid bars above sequence. Numbering is based on the bovine promoter sequence and an arrowhead indicates the transcriptional start site (+1). B, bovine; H, Human; C, chimp; D, dog; R, rat; M, mouse.

Matrix 2 yielded the greatest difference between the scores of competitors and non-competitors (Fig. 10). Possible scores ranged from -17.45 to 10.65, with the scores for the KLF15 protected sites ranging from 7.26 to 10.27. Oligos that were good competitors in EMSA all contained at least one binding site with a score > 5.68, whereas those that were poor competitors had maximal scores < 4.06. Using 1/2 of the difference between these scores as a cutoff (4.87), we analyzed the proximal promoters of *rhodopsin *(Fig. 8A) and *IRBP *(Fig. 8B) from human, chimp, dog, mouse and rat to identify potential KLF15 binding sites (Fig. 8; see also Additional file: 1). These analyses showed that the KR-a site was conserved across all mammalian *rhodopsin *promoters analyzed, and the KR-d site was present in all but the dog promoter, which contained an additional KLF15 binding site immediately 5' to KR-e. The other four KLF15 binding sites were conserved only in non-rodents, although one novel binding site located 3' to site KR-d was predicted in the mouse and rat promoters. In the *IRBP *promoters, KI-b and KI-c were conserved in bovine, human, chimp and dog, with an additional site predicted in the mouse promoter (5' to KI-a) and in bovine and dog (5' to KI-c, outside of the region analyzed by DNAse I footprinting).

### Effects of KLF15 on a minimal bovine rhodopsin promoter

**Figure 9:**
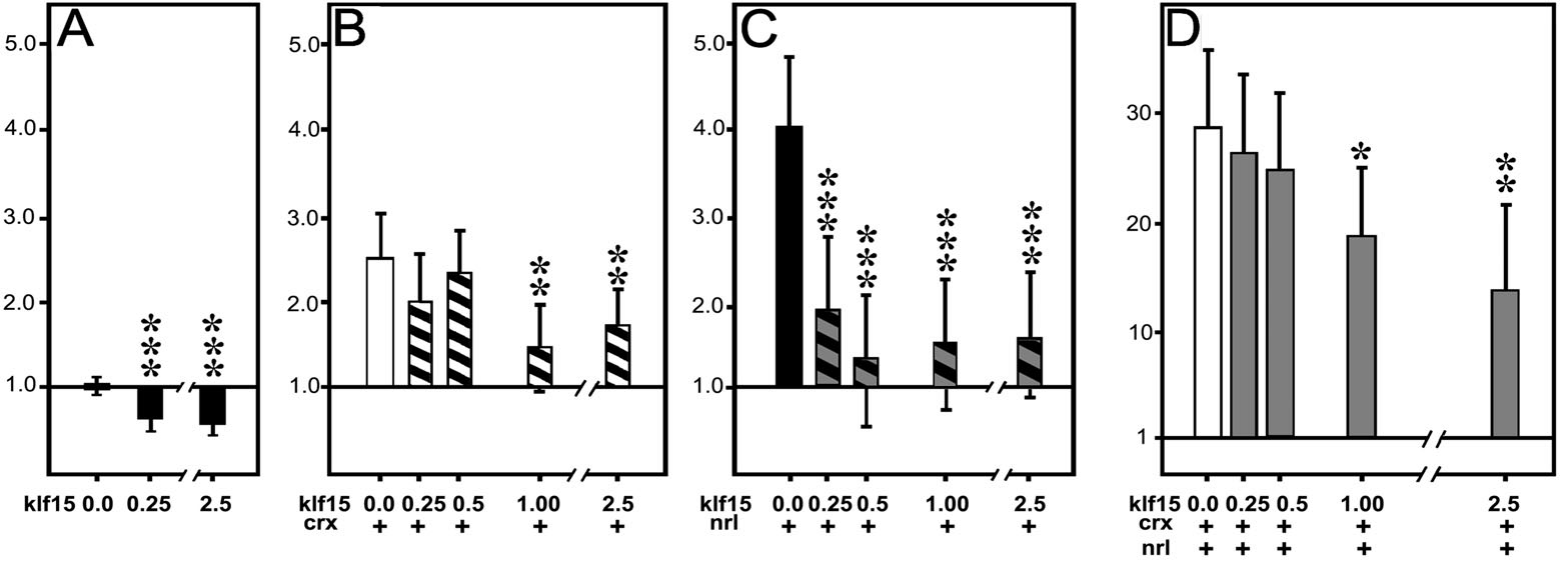
Effects of KLF15 on *rhodopsin *promoter. (A) 293 cells in 22 mm dishes were co-transfected with 2.5 μg of bovine rhodopsin (-130 to +70 bp)-luciferase fusion construct (bRho130-luc) together with the indicated amount (μg) of KLF15 expression vector. Luciferase activity (in relative light units) was corrected for transfection efficiency using β-GAL internal control. Bars show fold-change in relative luciferase expression compared to control transfections using expression vector lacking cDNA inserts. Error bars, 95% confidence interval. (B) same as (A) except 0.25 μg Crx expression vector was added. (C) Same as (A) except 0.25 μg Nrl expression vector was added. (D) Same as (A) except 0.25 μg Crx and 0.25 μg Nrl were added. Statistical differences from (A) control, (B) Crx alone; (C) Nrl alone; and (D) Crx+Nrl indicated by * p = 0.012, ** p < 0.008, *** p < 0.0001.

**Figure 10:**
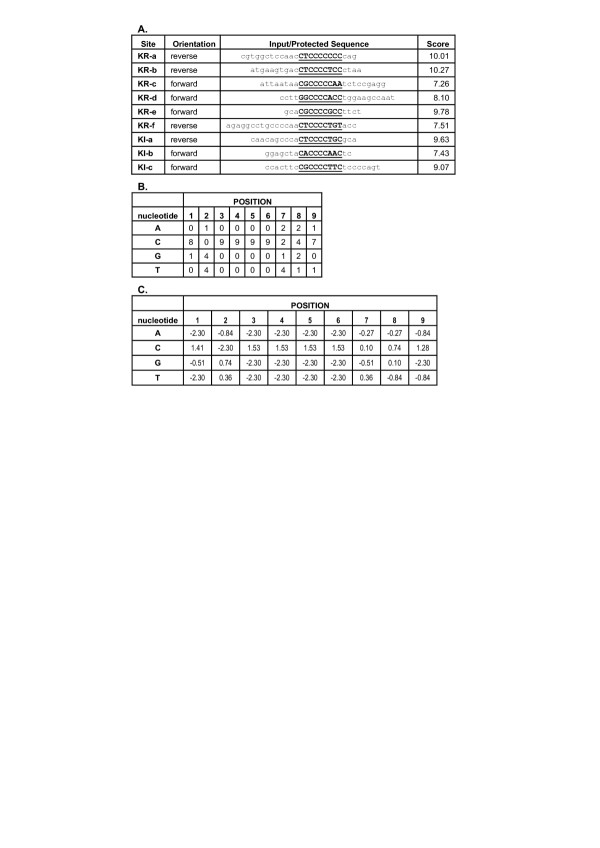
Analysis of KLF15 protected sequences in *Rhodopsin* and *IRBP* promoters using Target Explorer: Matrix 2: A. Alignment Matrix; B. Frequency Matrix; C. Scoring Matrix

We previously reported that KLF15 repressed transactivation of bRho225-luc, a promoter-luciferase reporter construct containing -225 to +70 bp of the bovine *rhodopsin *promoter [52] that contained the six KLF15 binding sites identified in DNAse I footprinting analysis. A smaller fragment of the *rhodopsin *proximal promoter (-130 to +70 bp) containing only four KLF15 binding sites, but lacking the KR-e/CRS-1 and KR-f sites, is still sufficient to drive expression of a reporter gene in primary cultures retinal cells from chick [36]. Using a luciferase reporter construct containing this smaller fragment of the *rhodopsin *promoter (bRho130-luc), the results of transient transfections of 293 cells were qualitatively similar to those previously reported using the bRho225-luc construct: KLF15 alone or in co-transfections with CRX and/or NRL resulted in statistically significant decreases in luciferase expression (Fig. 9). In co-transfections with CRX, high concentrations of KLF15 were more effective at reducing luciferase expression (Fig. 9B); in contrast, there was a relatively concentration-independent reduction (>50%) in luciferase expression in co-transfections with NRL at all concentrations of KLF15 tested (Fig. 9C). We compared the results of promoter transactivation assays using bRho130-luc with those previously obtained using bRho225-luc [52] and found that luciferase expression in transfections with KLF15 was consistently lower with bRho225-luc than with bRho130-luc, with the differences within the 95% confidence interval for KFL15+CRX (p = 0.0355) and within 90% confidence interval for KLF15 alone (p = 0.0879), KLF15+NRL (p = 0.0594) and KLF15+CRX+NRL (p = 0.0894).

## Discussion

We previously identified KLF15 as a transcriptional repressor of the *rhodopsin *and *IRBP *promoters *in vitro *and report here the characterization of KLF15's DNA binding properties. For these analyses, we analyzed protein binding specificity on bovine promoters using a truncated human KLF15-GST fusion protein containing the C-terminal DNA binding domain, but lacking the N-terminal repressor domain [52]. Since zinc-finger domains typically function as modular DNA binding motifs that can bind their target sequences independently of other protein domains [67,68], the binding specificity of the truncated KLF15 fusion protein is predicted to be similar, if not identical, to the full length protein. Previous studies showed that recombinant GST fusion proteins containing the full length human or rat KLF15 bound to an element in the CLC-K1 promoter [53] and we found that our truncated KLF15 fusion protein also bound the same element in EMSA (data not shown). The amino acid sequence of the zinc finger domains of bovine KLF15 differs from human, chimp, dog, mouse and rat at only a single amino acid residue (isoleucine to valine) and the flanking regions that were included in the KLF15-ZF fusion protein differ at only 11 amino acid residues between human and bovine KLF15. We would predict, therefore, that the DNA binding specificity would show only minor differences (if any) between these species. Supporting this prediction, the human KLF15-GST fusion protein shifted oligos containing either the bovine or human KLF15 consensus site. Using EMSA, we have confirmed that proteins present in retinal extracts can bind specifically to the KLF15 binding site. However, this result does not necessarily mean that the observed binding activity is attributable to KLF15. Other members of the KLF family recognize similar G/C rich binding motifs [54,57-59] and MAZ (myc-associated zinc-finger protein) can also recognize the KLF15 core binding site [53]. Thus, it remains a distinct possibility that, in addition to KLF15, there are other retinal proteins that can recognize this site.

Several of the KLF15 binding sites that we identified in DNAse I footprinting correspond to previously identified promoter elements. In EMSA, both retina-specific and ubiquitous nuclear proteins bind a 20 bp probe (-55 to -36) containing the Ret-4 element (including the KR-b site) in the *rhodopsin *promoter [24]. Interestingly, mutations in the G-rich sequence within the Ret-4 element that would be predicted to disrupt the KLF15 binding site eliminated binding by the ubiquitously expressed proteins [24]. In DNAse I footprinting assays of the bovine *rhodopsin *proximal promoter, nuclear extracts from bovine retina [21] and from Y79 and WERI retinoblastoma cells [69] protected the BAT-1 (-103 to -84) site. Two additional sites protected by bovine retinal proteins are Ret-1 (-138 to -126) and CRS-1 (-177 to -199) [16,20]. Three of these sites correspond to sites protected by KLF15 in our DNAse I footprinting, KR-6/Ret-4, KR-c/BAT-1 and KR-e/CRS-1. In addition to being protected by nuclear proteins from retinal extract, the CRS-1 site is protected by proteins present in nuclear extracts from non-retinal tissues [21]. KLF15 is expressed in the multiple tissues including the retina [52-54] and can repress *rhodopsin *promoter constructs in transient transfections [52]. Although we do not yet know the relative importance of these different KLF15 binding sites in regulation of target gene expression *in vivo*, our *in vitro *assays showed that KLF15 more effectively repressed a *rhodopsin *promoter construct (bRho225) containing six KLF15 binding sites (including KR-e/CRS-1) than a shorter promoter construct (bRho130) that contains only 4 (including the KR-b/Ret-4 and KR-c/BAT-1 sites), suggesting that both the proximal and distal sites influence KLF15's repressor activity.

The G-rich repressor element is located between -156 and -70 bp in *IRBP *promoter and overlaps the KI-c binding site for KLF15. Deletion of the G-rich element increases reporter gene expression in retinoblastoma cells [14,19,34] and results in inappropriate expression of GFP reporter constructs in non-retinal tissues in *Xenopus laevis *[28]. In DNAse I footprinting, this site is protected by proteins present in nuclear extracts from both retinoblastoma WERI-Rb1 and non-retinal HeLa cells [18]. Based on the ability of oligos containing the Sp1 consensus site to compete with the G-rich element in EMSA, it was proposed that Sp1 or Sp1-related proteins bind this site *in vivo *[19]. KLF15 represses transactivation of an *IRBP *promoter construct *in vitro*, is expressed in both retinal and non-retinal tissues, and is structurally and phylogenetically related to the Sp family of transcription factors [52], making it an attractive candidate to bind the G-rich repressor element *in vivo*.

Potential KLF15 binding sites corresponding to the CRS-1 and G-rich sites are present in the bovine, human, chimp and dog promoters. It was, however, somewhat surprising that no consensus KLF15 binding sites were identified at corresponding locations in the mouse or rat promoters. As the zinc-finger domains are identical between these species, it seems unlikely that the lack of identifiable binding sites at these locations reflects species-specific differences in KLF15's binding specificity. Although we cannot formally exclude this possibility, there are several possible alternative interpretations of this observation. The simplest explanation is that the location or number of KLF15 binding sites differs between species, possibly reflecting the accumulation of mutations during evolution. A comparison of well characterized promoters of 51 genes found substantial differences between human and mouse, with 32–40% of the binding sites identified in the human promoters containing sufficient sequence changes in the mouse promoters to render them non-functional [70]. In addition, despite our evidence showing that KLF15 binds the CRS-1 and G-rich repressor elements *in vitro*, there may be species-specific differences in the identity and/or sequence specificity of the transcription factor(s) that bind this site *in vivo*.

Changes in transcriptional regulation are thought to play a significant role in generating phenotypic differences between species [71] and there are clearly morphological and developmental differences between cows, primates, dogs and rodents. In the retina, differences include the time course of embryonic/fetal development and cellular differentiation, as well as the proportions and absolute numbers of different neuronal subtypes (e.g. rods vs. cones) present in the mature retina. Along with these morphological differences, there are species-specific variations in the regulation of photoreceptor-specific gene expression. In the bovine retina, transcripts for *rhodopsin*, *IRBP*, *arrestin*, rod α-*transducin *and rod α-*PDE *all begin to accumulate relatively late in retinal development, at the time when the first outer segments are detected [7]. In contrast, in mice and rats there is considerable variation in the developmental stage when different photoreceptor-specific genes are first expressed [8,9,72]. Interestingly, the CRS-1/KR-e site was initially proposed to play a role in repressing premature *rhodopsin *expression during retinal development, based on the observation that protection of the CRS-1 site by fetal bovine retinal proteins diminishes throughout development [21] in a temporal pattern that is coordinated with increases in *rhodopsin *transcription and mRNA accumulation [9]. Further emphasizing the extent of the differences between species, a large scale analysis of expressed genes in the mouse retina found that out of nearly 2000 known genes that could be assigned map locations in the mouse genome, 25% had no identifiable human ortholog [73]. Although the identification and characterization of conserved genes and regulatory mechanisms has provided significant contributions to our understanding of retinal development and disease, there are likely to be novel and potentially valuable insights that can be obtained through comparative analysis of the basis of species-specific differences.

Several KLF proteins have been shown to bind a core CACCC element [57-59] and Gray and colleagues [54] demonstrated that 5'-CACCC-3' is sufficient for KLF15 binding in EMSA. A single zinc finger typically makes contact with 3 adjacent bases [67] therefore the three zinc-fingers of KLF15 would be predicted to have a 9 bp binding site, consistent with our EMSA results. We found that mutations outside of the 9 bp consensus site had no effect on DNA binding in EMSA; however, when we used TargetExplorer to re-analyze the KLF15 protected sequences identified in DNAse I footprinting, making no *a priori *assumptions as to the actual size of the consensus binding site, a longer 12–13 bp consensus sequence was identified. The functional significance of the extended consensus site is unclear, but it is interesting to note that the KR-b, KR-c and KR-d sites respectively overlapped the Ret-4, BAT-1 and Ret-1 sites, all containing known binding sites for homeodomain containing proteins including CRX [30,31]. The KR-b site is located at the 3' end of the Ret-4 site within a G-rich sequence is a binding site for nuclear proteins present in both retinal and non-retinal tissues [24]. The highly conserved KR-d site is within a GC-rich sequence that functions to facilitate binding of retinal nuclear proteins to the adjacent Ret-1 site [15]. One interpretation is that KLF15 binding sites are preferentially located adjacent to binding sites for other transcription factors, possibly CRX or other OTX-related factors. In EMSA using total retinal proteins, mutation of the KLF15 binding site increased binding of some retinal proteins to the Δ10 oligo, as shown by the increased intensity of KLF15-independent bands (Fig. 5). As mutation of the KLF15 binding site does not alter the core homeodomain binding site present in these oligos, homeodomain proteins (e.g. CRX; RX/RAX; OTX2) present in retinal extracts likely bind to both the wild-type and mutant oligos. Although mutation of the KLF15 site may have inadvertently created a novel protein binding site, several of the bands showing increased intensity with the Δ10 oligo were also present using the wild-type bRho29 oligo, raising the possibility that occupancy of the KLF15 site can affect protein/DNA interactions of other DNA binding proteins. The proximity of KLF15 and CRX binding sites and our findings that (1) KLF15 can repress transactivation of the *rhodopsin *promoter by CRX *in vitro *and (2) both KFL15 and CRX are expressed in non-photoreceptor cells in the inner nuclear layer [52] are consistent with a possible *in vivo *role for KLF15 in repressing inappropriate activation of the *rhodopsin *promoter by CRX in non-photoreceptor cells.

A similar correlation between KLF15 and CRX binding sites was not observed in the IRBP promoter. However, the KI-c site/G-rich repressor element in the bovine IRBP promoter is adjacent to a CpG dinucleotide (-115) that becomes hypomethylated specifically in retinal cells at the time *IRBP *expression is first detected [74,75]. The G-rich repressor element is bound by proteins present in non-retinal tissues where the CpG dinucleotide remains methylated; therefore, it is tempting to speculate that KLF15 may participate in regulating methylation at this site. Other KLF genes are known to regulate chromatin remodeling. The transcriptional activator KLF1/EKLF recruits a SWI/SNF-related chromatin remodeling complex that is thought to be involved in regulation of the locus control region of the β-globin gene [76]. The transcriptional repressor KLF14/BTEB3 interacts with a co-repressor (mSin3A) and the histone deacetylase protein HDAC-1 though repressor domains within the N-terminus to mediate transcriptional repression through chromatin remodeling [77]. Interestingly, in our footprinting analysis of the *rhodopsin *promoter we observed several sites of increased DNAse I hypersensitivity adjacent to KLF15 protected regions, possibly reflecting alterations in DNA configuration and potentially lending additional support for involvement of KLF15 in chromatin remodeling.

## Conclusion

The goal of these studies was to determine the binding characteristics and specificity of KLF15 and define the KLF15 binding sites in the *Rho *and *IRBP *promoters in order to gain further insight into the mechanism of KLF15-mediated regulation of retinal gene expression. We found that KLF15 binds to multiple 9 bp consensus sites in the *rhodopsin *and *IRBP *promoters including the previously identified CRS-1 and G-rich repressor elements. The presence of multiple KLF15 binding sites suggests that transcriptional repression is likely to play an important role in regulation of photoreceptor-specific genes. Based on the known expression of KLF15 in retinal and non-retinal tissues and the absence of KLF15 expression in photoreceptors, we hypothesize an *in vivo *role for KLF15 in repressing photoreceptor-specific gene expression in non-photoreceptor cells.

## Methods

### Protein isolation

To generate KLF15-ZF-GST, the 3' end of the human KLF15 coding region (369 bp) and the stop codon was amplified from pCRII-hKLF15 [52] using a combination of Taq (Invitrogen; Carlsbad, California) and PFU polymerase (Promega; Madison, WI), subcloned into the BamHI/EcoRI sites of pGEX (Amersham/Pharmacia; Piscataway, NJ) and confirmed by sequencing. KLF15-ZF fusion protein with an N-terminal, glutathione-S-transferase (GST) tag was purified from whole cell lysates of IPTG-induced (0.4 mM) *E. coli *(BL21S; Invitrogen; Carlsbad, California) using sepharose 4B (Amersham/Pharmacia; Piscataway, NJ) according to maunfacturer's instructions. Purified protein was quantified using bicinchoninic acid protein assay kit (BCA; Sigma; St. Louis, MO) and analyzed using standard 10% SDS-polyacrylamide gel electrophoresis and Western blot with anti-GST antibodies (Amersham/Pharmacia; Piscataway, NJ) diluted 1:8000.

Mice used for protein isolation were handled in accordance with the Association for Research in Vision and Ophthalmology Statement for the Use of Animals and methods were approved by the Animal Care and Use Committee of Johns Hopkins University. For total protein extracts, retinas were dissected from adult mice and placed directly into ice cold PBS containing protease inhibitors (CompleteMini Tablets, EDTA-free; Roche, Indianapolis, IN), sonicated for 10–15 seconds (Setting 5, 50% Duty Cycle) using a Branson Model 250 Sonifier (Branson Ultrasonics Corp., Danbury, CT) with a micro-tip, quantified by BCA and stored at -80C.

### Electrophoretic mobility shift assays (EMSA)

EMSA assays followed previously published protocols [24,78] and used a binding buffer optimized for the KLF15-ZF-GST fusion protein (4 mM HEPES, 5 mM EGTA; 100 mM KCl, 0.25 mM ZnCl_2_; 0.02% NP40; 0.1 M DTT; 4% glycerol). For analysis of Zn^+2 ^specificity, ZnCl_2 _was eliminated from the binding buffer except as indicated. Probes with 5'-GG dinucleotide overhangs were generated by annealing single-stranded oligos with their reverse complement and end-labeled with ^32^P-dCTP using Klenow fragment of DNA polymerase (New England Biolabs, Beverly MA). Cold competitors were end-filled with Klenow using cold dCTP. Binding reactions were incubated for 90 to 120 minutes on ice; for supershift experiments antibodies against GST (Amersham/Pharmacia; Piscataway, NJ) or KLF15 C-terminal peptide [a generous gift of S. Uchida; [53]] were added after an initial 60 minute incubation. Following polyacrylamide gel electrophoresis on a 5% non-denaturing gel, dried gels were exposed to storage phosphor screens; peaks for each band and DLU (Digital Light Units) for the areas under the peaks were determined using Optiquant software (Packard Instruments). For each lane, the percent of oligo shifted was calculated as [(DLU shifted band/ total DLU) × 100]. For competition assays, "percent competed" was calculated as the difference between the percent oligo shifted without competitor and the percent oligo shifted with competitor.

### DNAse I footprint analysis

DNAse I footprinting followed previously published procedures [24,78] using ^33^P-end-labeled promoter fragments generated by PCR, KLF15-ZF-GST or GST fusion proteins and EMSA binding buffer optimized for KLF15-ZF. For bovine *Rho *promoter fragments, the template was a plasmid containing the bovine upstream *Rho *promoter (-2174 to +32 bp) [36]; for bovine *IRBP *promoter fragment (-300 to +132 bp), the template was a plasmid containing the corresponding region of the bovine *IRBP *promoter [30]. Primers used for PCR amplification of promoter fragments were as follows: for bovine *Rho *(-225 to +70 bp): forward: 5'-AGGCCTCTGCTCTTTCCC-3'; reverse: 5'-CGCCGGCGGCGCGAACCCG-3'; for bovine *Rho *(-315 to -31 bp): forward: 5'-AGAGGGAAGTGGGCCTAGAG-3', reverse 5'- GAAGTGACCTCCCCTCCCTA-3'; for bovine *IRBP*: forward: 5'-CAGATGAGACCCCAACATAC-3'; reverse: 5'-ACAGAAGCTCTCTTGACACC-3'. The amount of fusion protein used varied from 16 to 250 ng per reaction and DNAse I digestions were carried out at room temperature for 1 minute.

### Consensus sequence analysis

To identify consensus sequences for protein binding sites, sequences protected in DNAse I footprinting were analyzed using Target Explorer [66], a program that uses CONSENSUS (version 6c) and WCONSENSUS (version 5c) to determine consensus patterns in unaligned sequences using an algorithm based on a matrix representation of a consensus pattern. WCONSENSUS determines the width of the pattern being sought without *a priori *knowledge of the length of the consensus sequence.

### Transient transfections

Promoter transactivation assays used transient transfections of human embryonic kidney cells (293) with expression vectors, hKLF15-pcDNA3.1 [52], Crx-pcDNA3.1 [30], Nrl-pED (gift of A. Swaroop [35]) and luciferase reporter plasmid bRho130-luc [36] following previously published methods [30,52]. The total amount (μg) of DNA in each plate was kept constant by addition of the corresponding expression vector(s) lacking a cDNA insert. Basal activity of each reporter construct in 293 cells was defined as the relative luciferase expression in control plates transfected with reporter constructs and "empty" expression vectors. For each experiment, at least two independent transfections were performed on separate days, and for each combination of expression vectors tested, at least 4 DNA precipitates prepared with each precipitate divided equally to transfect two plates for a total of≥8 transfections per condition.

### Statistical analyses

For each condition the fold change in relative luciferase relative to control transfections was calculated and analyzed as previously described [52]. Statistical analysis used linear mixed models and comparisons of bRho130-luc and bRho225-luc reporter constructs used Wilcoxon Two Sample test (SAS; Cary, NC).

## List of abbreviations

ATXN7, ataxin-7; BAF, barrier to autointegration factor 1; BTEB3, basic transcription element binding protein 3; Clc-K, kidney-specific chloride channel; CRX, cone-rod homeobox; DTT, dithiothreitol; EDTA, ethylenediaminetraacetic acid; EGTA, O,O'-bis(2-aminoethyl)ethyleneglycol-N,N,N',N'-tetraacetic acid; EKLF, erythroid-specific *Krüppel*-like factor; ERX, empty spiracles-related homeobox; FIZ-1, Flt3 interacting zinc finger protein 1; GST, glutathione-S-transferase; HEPES, N-2-hydroxyethylpiperazine-N'-2-ethanesulfonic acid; IPTG, isopropyl-β-D-thiogalactopyranoside; KLF, *Krüppel*-like factor; NRL, neural retina leucine zipper; OTX, orthodenticle-related homeobox; QRX, Q50-type retinal homeobox; Rbp3, retinoid binding protein 3; RX/RAX, retina and anterior neural fold homeobox.

## Authors' contributions

DCO participated in the experimental design, generated and purified fusion proteins, carried out EMSA, DNAse I footprinting, transient transfections, consensus sequence analysis, promoter alignments and drafted the manuscript. HL performed all statistical analyses and contributed to the interpretation of the transfection data; YL isolated the KLF15 clone and contributed to the conceptual basis of the study; DJZ conceived the study, participated in experimental design and data interpretation and helped to draft the manuscript. All authors read and approved the final manuscript.

## Supplementary Material

Additional File 1 Supplemental Data Files (Otteson SupplementalData.doc) containing full results of analysis of KFL15 binding sites using Target Explorer. Included in this data set are: 1. Sequences from bovine rhodopsin and IRBP promoters protected by KLF15-gst fusion proteins in DNAseI footprint analysis used as "training set" for Target Explorer. 2. List of top matrices for **KLF15_9bpsite_otteson **binding site. (sorted by information content): Assume 9 bp binding site and 60% AT/40% GC in genome. 3. Analysis to determine cutoff score for identification of KLF15 binding sites. Sequences and scores for bRho29, hRho29 and bRho29-mutations oligonucleotides used in competitive EMSA. Lists of putative binding sites in these oligos identified using Matrices 1–4: cut-off score = 1 indicating good and poor competitors. 4. List of top matrices for **KLF15: Assume unknown **binding site length and 60% T/40% GC in genome. 5. Analysis to determine cutoff score for identification of KLF15 binding sites assuming unknown binding site length. Sequences and scores for bRho29, hRho29 and bRho29-mutations oligonucleotides used in competitive EMSA. Lists of putative binding sites in these oligos identified using Matrices 1–4: cut-off score = 1 indicating good and poor competitors. 6. Identification of potential KLF15 binding sites in rhodopsin and IRBP promoters using matrix 2 with 9 bp binding site (cut-off score 4.87). Promoter sequences analyzed. Predicted binding sites Click here for file
