# Supplementary material for: Zinc-finger domains of the transcriptional repressor KLF15 bind multiple sites in rhodopsin and IRBP promoters including the CRS-1 and G-rich repressor elements
Source: BMC Mol Biol. 2005 Jun 17;6:15. doi: 10.1186/1471-2199-6-15 (PMC1182371; doi:10.1186/1471-2199-6-15)
Supplement: Additional File 1 — Supplemental Data Files (Otteson SupplementalData.doc) containing full results of analysis of KFL15 binding sites using Target Explorer. Included in this data set are: 1. Sequences from bovine rhodopsin and IRBP promoters protected by KLF15-gst fusion proteins in DNAseI footprint analysis used as "training set" for Target Explorer. 2. List of top matrices for KLF15_9bpsite_otteson binding site. (sorted by information content): Assume 9 bp binding site and 60% AT/40% GC in genome. 3. Analysis to determine cutoff score for identification of KLF15 binding sites. Sequences and scores for bRho29, hRho29 and bRho29-mutations oligonucleotides used in competitive EMSA. Lists of putative binding sites in these oligos identified using Matrices 1–4: cut-off score = 1 indicating good and poor competitors. 4. List of top matrices for KLF15: Assume unknown binding site length and 60% T/40% GC in genome. 5. Analysis to determine cutoff score for identification of KLF15 binding sites assuming unknown binding site length. Sequences and scores for bRho29, hRho29 and bRho29-mutations oligonucleotides used in competitive EMSA. Lists of putative binding sites in these oligos identified using Matrices 1–4: cut-off score = 1 indicating good and poor competitors. 6. Identification of potential KLF15 binding sites in rhodopsin and IRBP promoters using matrix 2 with 9 bp binding site (cut-off score 4.87). Promoter sequences analyzed. Predicted binding sites [file 1471-2199-6-15-S1.doc]

1. Sequences from bovine Rhodopsin and IRBP promoters protected by KLF15-gst fusion proteins in DNAseI footprint analysis used as “training set” for Target Explorer.

sites

>KR-a_rev

CGTGGCTCCAACCTCCCCCCCCAG

>KR-b_rev

ATGAAGTGACCTCCCCTCCCTAA

>KR-c_for

ATTAATAACGCCCCCAATCTCCGAGG

>KR-d_for

CCTTGGCCCCACCTGGAAGCCAAT

>KR-e_for

GCACGCCCCGCCTTCT

>KR-f_rev

AGAGGCCTGCCCCAACTCCCCTGTACC

>KIa_rev

CAACAGCCCACTCCCCTGCGCA

>KIb_for

GGAGCTACACCCCAACTC

>KI-c_for

CCACTTCCGCCCCTTCTCCCCAGT

2. LIST OF TOP MATRICES FOR **KLF15_9bpsite_otteson** binding site

(sorted by information content)

Assume 9 bp binding site

Assume 60% AT/40%GC in genome

sequence 1: KR-a_rev

sequence 2: KR-b_rev

sequence 3: KR-c_for

sequence 4: KR-d_for

sequence 5: KR-e_for

sequence 6: KR-f_rev

sequence 7: KIa_rev

sequence 8: KIb_for

sequence 9: KI-c_for

--------------------------------------------------------------------------------

**MATRIX 1**

information content = 7.52829

expected frequency = 2.17559E-15

**alignment matrix**

A 0 1 0 0 0 0 3 3 1

C 7 0 9 9 9 9 2 4 8

G 1 5 0 0 0 0 1 1 0

T 1 3 0 0 0 0 3 1 0

**sequence alignment**

seq position motif

1 13 C T C C C C C C C

2 11 C T C C C C T C C

3 9 C G C C C C C A A

4 5 G G C C C C A C C

5 4 C G C C C C G C C

6 8 T G C C C C A A C

7 11 C T C C C C T G C

8 8 C A C C C C A A C

9 8 C G C C C C T T C

--------------------------------------------------------------------------------

**MATRIX 2**

information content = 7.47974

expected frequency = 3.68171E-15

**alignment matrix**

A 0 1 0 0 0 0 2 2 1

C 8 0 9 9 9 9 2 4 7

G 1 4 0 0 0 0 1 2 0

T 0 4 0 0 0 0 4 1 1

**sequence alignment**

seq position motif

1 13 C T C C C C C C C

2 11 C T C C C C T C C

3 9 C G C C C C C A A

4 5 G G C C C C A C C

5 4 C G C C C C G C C

6 16 C T C C C C T G T

7 11 C T C C C C T G C

8 8 C A C C C C A A C

9 8 C G C C C C T T C

-------------------------------------------------------------------------------

**MATRIX 3**

information content = 7.47229

expected frequency = 3.99045E-15

**alignment matrix**

A 0 1 0 0 0 0 3 2 1

C 8 0 9 9 9 9 2 4 6

G 1 3 0 0 0 0 1 3 0

T 0 5 0 0 0 0 3 0 2

**sequence alignment**

seq position motif

1 13 C T C C C C C C C

2 11 C T C C C C T C C

3 9 C G C C C C C A A

4 5 G G C C C C A C C

5 4 C G C C C C G C C

6 16 C T C C C C T G T

7 11 C T C C C C T G C

8 8 C A C C C C A A C

9 16 C T C C C C A G T

--------------------------------------------------------------------------------

**MATRIX 4**

information content = 7.40318

expected frequency = 8.41299E-15

**alignment matrix**

A 0 2 5 0 1 0 0 0 0

C 7 4 0 8 2 9 9 8 9

G 1 0 0 1 3 0 0 0 0

T 1 3 4 0 3 0 0 1 0

**sequence alignment**

seq position motif

1 12 C C T C C C C C C

2 10 C C T C C C C T C

3 6 T A A C G C C C C

4 2 C T T G G C C C C

5 1 G C A C G C C C C

6 13 C A A C T C C C C

7 8 C C A C T C C C C

8 5 C T A C A C C C C

9 13 C T T C T C C C C

---------------------------------------------------------------------------------------------------------

3. Analysis to determine cutoff score for identification of KLF15 binding sites

Scores for bRho29, hRho29, bRho29-mutations oligonucleotides used in competitive EMSA

>bRho29

TTAATAACGCCCCCAATCTCCGAGGTGC

>hRho29

TTATGAACACCCCCAATCTCCCAGATGC

>bRho29D1

GGCATAACGCCCCCAATCTCCGAGGTGC

>bRho29D4

TTACGCACGCCCCCAATCTCCGAGGTGC

>bRho29D7

ttaatacatcccccaatctccgaggtgc

>bRho29D10

ttaataacgaaaccaatctccgaggtgc

>bRho29D13

ttaataacgcccaacatctccgaggtgc

>bRho29D16

TTAATAACGCCCCCACGATCCGAGGTGC

>bRho29D19

TTAATAACGCCCCCAATCGAAGAGGTGC

>bRho29D22

TTAATAACGCCCCCAATCTCCTGTGTGC

>bRho29D25

TTAATAACGCCCCCAATCTCCGAGTGTC

>bRho29D11

ttaataacgcacccaatctccgagtgtc

>bRho29D7+D13

ttaatacatcccaacatctccgaggtgc

>bRho29D8_13

ttaataaagcccacaatctccgaggtgc

>IRBP1

ggacaggattaaaggcttactggag

>IRBP2

ggacttgtcagggccttta

#### List of putative binding sites for matrix 1: KLF15_9bpsite_otteson1 (cut-off score = 1)

Good competitors CAPS/underscored; poor competitors, lowercase.

Highest score for each oligo in bold, underlined

Highest score for competitor: 9.97

Lowest score for compeitor: 5.92

Highest score for non-competitor: 4.53

Difference: 1.39

|  | seq name |  | TF name | BS dir | BS seq | BS pos | BS score |
| --- | --- | --- | --- | --- | --- | --- | --- |
| 1 | bRho29 |  | KLF15_9bpsite_otteson1 | for | AACGCCCCC | 6 | 1.40 |
| **2** | **bRho29** |  | **KLF15_9bpsite_otteson1** | **for** | **CGCCCCCAA** | **8** | **7.72** |
| 3 | bRho29 |  | KLF15_9bpsite_otteson1 | for | GCCCCCAAT | 9 | 1.21 |
| 4 | bRho29 |  | KLF15_9bpsite_otteson1 | for | CCCCCAATC | 10 | 1.94 |
| 5 | hRho29 |  | KLF15_9bpsite_otteson1 | for | AACACCCCC | 6 | 1.40 |
| **6** | **hRho29** |  | **KLF15_9bpsite_otteson1** | **for** | **CACCCCCAA** | **8** | **5.92** |
| 7 | hRho29 |  | KLF15_9bpsite_otteson1 | for | CCCCCAATC | 10 | 1.94 |
| 8 | bRho29D1 |  | KLF15_9bpsite_otteson1 | for | AACGCCCCC | 6 | 1.40 |
| **9** | **bRho29D1** |  | **KLF15_9bpsite_otteson1** | **for** | **CGCCCCCAA** | **8** | **7.72** |
| 10 | bRho29D1 |  | KLF15_9bpsite_otteson1 | for | GCCCCCAAT | 9 | 1.21 |
| 11 | bRho29D1 |  | KLF15_9bpsite_otteson1 | for | CCCCCAATC | 10 | 1.94 |
| 12 | bRho29D4 |  | KLF15_9bpsite_otteson1 | for | CGCACGCCC | 4 | 2.95 |
| 13 | bRho29D4 |  | KLF15_9bpsite_otteson1 | for | CACGCCCCC | 6 | 4.98 |
| **14** | **bRho29D4** |  | **KLF15_9bpsite_otteson1** | **for** | **CGCCCCCAA** | **8** | **7.72** |
| 15 | bRho29D4 |  | KLF15_9bpsite_otteson1 | for | GCCCCCAAT | 9 | 1.21 |
| 16 | bRho29D4 |  | KLF15_9bpsite_otteson1 | for | CCCCCAATC | 10 | 1.94 |
| 17 | bRho29D7 |  | KLF15_9bpsite_otteson1 | for | catccccca | 7 | 2.73 |
| **18** | **bRho29D7** |  | **KLF15_9bpsite_otteson1** | **for** | **atcccccaa** | **8** | **3.28** |
| 19 | bRho29D7 |  | KLF15_9bpsite_otteson1 | for | cccccaatc | 10 | 1.94 |
| **20** | **bRho29D13** |  | **KLF15_9bpsite_otteson1** | **for** | **cgcccaaca** | **8** | **4.53** |
| 21 | bRho29D16 |  | KLF15_9bpsite_otteson1 | for | AACGCCCCC | 6 | 1.40 |
| **22** | **bRho29D16** |  | **KLF15_9bpsite_otteson1** | **for** | **CGCCCCCAC** | **8** | **9.97** |
| 23 | bRho29D16 |  | KLF15_9bpsite_otteson1 | for | GCCCCCACG | 9 | 1.85 |
| 24 | bRho29D19 |  | KLF15_9bpsite_otteson1 | for | AACGCCCCC | 6 | 1.40 |
| **25** | **bRho29D19** |  | **KLF15_9bpsite_otteson1** | **for** | **CGCCCCCAA** | **8** | **7.72** |
| 26 | bRho29D19 |  | KLF15_9bpsite_otteson1 | for | GCCCCCAAT | 9 | 1.21 |
| 27 | bRho29D19 |  | KLF15_9bpsite_otteson1 | for | CCCCCAATC | 10 | 1.94 |
| 28 | bRho29D19 |  | KLF15_9bpsite_otteson1 | rev | GAAGAGGTG | 19 | 3.40 |
| 29 | bRho29D22 |  | KLF15_9bpsite_otteson1 | for | AACGCCCCC | 6 | 1.40 |
| **30** | **bRho29D22** |  | **KLF15_9bpsite_otteson1** | **for** | **CGCCCCCAA** | **8** | **7.72** |
| 31 | bRho29D22 |  | KLF15_9bpsite_otteson1 | for | GCCCCCAAT | 9 | 1.21 |
| 32 | bRho29D22 |  | KLF15_9bpsite_otteson1 | for | CCCCCAATC | 10 | 1.94 |
| 33 | bRho29D25 |  | KLF15_9bpsite_otteson1 | for | AACGCCCCC | 6 | 1.40 |
| **34** | **bRho29D25** |  | **KLF15_9bpsite_otteson1** | **for** | **CGCCCCCAA** | **8** | **7.72** |
| 35 | bRho29D25 |  | KLF15_9bpsite_otteson1 | for | GCCCCCAAT | 9 | 1.21 |
| 36 | bRho29D25 |  | KLF15_9bpsite_otteson1 | for | CCCCCAATC | 10 | 1.94 |
| **37** | **bRho29D11** |  | **KLF15_9bpsite_otteson1** | **for** | **cgcacccaa** | **8** | **3.89** |
| 38 | bRho29D11 |  | KLF15_9bpsite_otteson1 | for | cacccaatc | 10 | 3.40 |
| **39** | **bRho29D7+D13** |  | **KLF15_9bpsite_otteson1** | **for** | **catcccaac** | **7** | **4.34** |
| **40** | **IRBP2** |  | **KLF15_9bpsite_otteson1** | **rev** | **gtcagggcc** | **7** | **3.74** |

Good competitors CAPS; poor competitors, lowercase.

Highest score for each oligo in bold, underlined

Highest score for competitor: 9.38

Lowest score for compeitor: 5.68

Highest score for non-competitor: 4.01

Difference: 1.67

|  | seq name | TF name | BS dir | BS seq | BS pos | BS score |
| --- | --- | --- | --- | --- | --- | --- |
| 1 | bRho29 | klf15_60_40_9bpmatrix2 | for | AACGCCCCC | 6 | 1.27 |
| **2** | **bRho29** | **klf15_60_40_9bpmatrix2** | **for** | **CGCCCCCAA** | **8** | **7.26** |
| 3 | bRho29 | klf15_60_40_9bpmatrix2 | for | GCCCCCAAT | 9 | 1.93 |
| 4 | bRho29 | klf15_60_40_9bpmatrix2 | for | CCCCCAATC | 10 | 1.57 |
| 5 | bRho29 | klf15_60_40_9bpmatrix2 | rev | TCCGAGGTG | 19 | 1.61 |
| 6 | hRho29 | klf15_60_40_9bpmatrix2 | for | AACACCCCC | 6 | 1.27 |
| **7** | **hRho29** | **klf15_60_40_9bpmatrix2** | **for** | **CACCCCCAA** | **8** | **5.68** |
| 8 | hRho29 | klf15_60_40_9bpmatrix2 | for | CCCCCAATC | 10 | 1.57 |
| 9 | hRho29 | klf15_60_40_9bpmatrix2 | for | CTCCCAGAT | 18 | 2.44 |
| 10 | bRho29D1 | klf15_60_40_9bpmatrix2 | for | AACGCCCCC | 6 | 1.27 |
| **11** | **bRho29D1** | **klf15_60_40_9bpmatrix2** | **for** | **CGCCCCCAA** | **8** | **7.26** |
| 12 | bRho29D1 | klf15_60_40_9bpmatrix2 | for | GCCCCCAAT | 9 | 1.93 |
| 13 | bRho29D1 | klf15_60_40_9bpmatrix2 | for | CCCCCAATC | 10 | 1.57 |
| 14 | bRho29D1 | klf15_60_40_9bpmatrix2 | rev | TCCGAGGTG | 19 | 1.61 |
| 15 | bRho29D4 | klf15_60_40_9bpmatrix2 | for | CGCACGCCC | 4 | 2.73 |
| 16 | bRho29D4 | klf15_60_40_9bpmatrix2 | for | CACGCCCCC | 6 | 4.98 |
| **17** | **bRho29D4** | **klf15_60_40_9bpmatrix2** | **for** | **CGCCCCCAA** | **8** | **7.26** |
| 18 | bRho29D4 | klf15_60_40_9bpmatrix2 | for | GCCCCCAAT | 9 | 1.93 |
| 19 | bRho29D4 | klf15_60_40_9bpmatrix2 | for | CCCCCAATC | 10 | 1.57 |
| 20 | bRho29D4 | klf15_60_40_9bpmatrix2 | rev | TCCGAGGTG | 19 | 1.61 |
| 21 | bRho29D7 | klf15_60_40_9bpmatrix2 | for | catccccca | 7 | 2.86 |
| **22** | **bRho29D7** | **klf15_60_40_9bpmatrix2** | **for** | **atcccccaa** | **8** | **3.17** |
| 23 | bRho29D7 | klf15_60_40_9bpmatrix2 | for | cccccaatc | 10 | 1.57 |
| 24 | bRho29D7 | klf15_60_40_9bpmatrix2 | rev | tccgaggtg | 19 | 1.61 |
| **25** | **bRho29D10** | **klf15_60_40_9bpmatrix2** | **rev** | **tccgaggtg** | **19** | **1.61** |
| **26** | **bRho29D13** | **klf15_60_40_9bpmatrix2** | **for** | **cgcccaaca** | **8** | **4.07** |
| 27 | bRho29D13 | klf15_60_40_9bpmatrix2 | rev | tccgaggtg | 19 | 1.61 |
| 28 | bRho29D16 | klf15_60_40_9bpmatrix2 | for | AACGCCCCC | 6 | 1.27 |
| **29** | **bRho29D16** | **klf15_60_40_9bpmatrix2** | **for** | **CGCCCCCAC** | **8** | **9.38** |
| 30 | bRho29D16 | klf15_60_40_9bpmatrix2 | for | GCCCCCACG | 9 | 1.48 |
| 31 | bRho29D16 | klf15_60_40_9bpmatrix2 | rev | TCCGAGGTG | 19 | 1.61 |
| 32 | bRho29D19 | klf15_60_40_9bpmatrix2 | for | AACGCCCCC | 6 | 1.27 |
| **33** | **bRho29D19** | **klf15_60_40_9bpmatrix2** | **for** | **CGCCCCCAA** | **8** | **7.26** |
| 34 | bRho29D19 | klf15_60_40_9bpmatrix2 | for | GCCCCCAAT | 9 | 1.93 |
| 35 | bRho29D19 | klf15_60_40_9bpmatrix2 | for | CCCCCAATC | 10 | 1.57 |
| 36 | bRho29D19 | klf15_60_40_9bpmatrix2 | rev | GAAGAGGTG | 19 | 3.66 |
| 37 | bRho29D22 | klf15_60_40_9bpmatrix2 | for | AACGCCCCC | 6 | 1.27 |
| **38** | **bRho29D22** | **klf15_60_40_9bpmatrix2** | **for** | **CGCCCCCAA** | **8** | **7.26** |
| 39 | bRho29D22 | klf15_60_40_9bpmatrix2 | for | GCCCCCAAT | 9 | 1.93 |
| 40 | bRho29D22 | klf15_60_40_9bpmatrix2 | for | CCCCCAATC | 10 | 1.57 |
| 41 | bRho29D25 | klf15_60_40_9bpmatrix2 | for | AACGCCCCC | 6 | 1.27 |
| **42** | **bRho29D25** | **klf15_60_40_9bpmatrix2** | **for** | **CGCCCCCAA** | **8** | **7.26** |
| 43 | bRho29D25 | klf15_60_40_9bpmatrix2 | for | GCCCCCAAT | 9 | 1.93 |
| 44 | bRho29D25 | klf15_60_40_9bpmatrix2 | for | CCCCCAATC | 10 | 1.57 |
| **45** | **bRho29D11** | **klf15_60_40_9bpmatrix2** | **for** | **cgcacccaa** | **8** | **3.43** |
| 46 | bRho29D11 | klf15_60_40_9bpmatrix2 | for | cacccaatc | 10 | 3.03 |
| **47** | **bRho29D7+D13** | **klf15_60_40_9bpmatrix2** | **for** | **catcccaac** | **7** | **3.60** |
| 48 | bRho29D7+D13 | klf15_60_40_9bpmatrix2 | rev | tccgaggtg | 19 | 1.61 |
| **49** | **bRho29D8_13** | **klf15_60_40_9bpmatrix2** | **rev** | **tccgaggtg** | **19** | **1.61** |
| **50** | **IRBP2** | **klf15_60_40_9bpmatrix2** | **rev** | **gtcagggcc** | **7** | **3.02** |

#### ------------------------------------------------------------------------------------------------------

#### List of putative binding sites for matrix 3: KLF15_9bpsite_otteson3a (cut-off score 1)

Good competitors CAPS; poor competitors, lowercase.

Highest score for each oligo in bold, underlined

Highest score for competitor: 8.96

Lowest score for compeitor: 5.68

Highest score for non-competitor: 4.17

Difference: 1.51

|  | seq name | TF name | BS dir | BS seq | BS pos | BS score |
| --- | --- | --- | --- | --- | --- | --- |
| 1 | bRho29 | KLF15_9bpsite_otteson3a | for | AACGCCCCC | 6 | 1.12 |
| **2** | **bRho29** | **KLF15_9bpsite_otteson3a** | **for** | **CGCCCCCAA** | **8** | **6.99** |
| 3 | bRho29 | KLF15_9bpsite_otteson3a | for | GCCCCCAAT | 9 | 2.87 |
| 4 | bRho29 | KLF15_9bpsite_otteson3a | rev | TCCGAGGTG | 19 | 1.98 |
| 5 | hRho29 | KLF15_9bpsite_otteson3a | for | AACACCCCC | 6 | 1.12 |
| **6** | **hRho29** | **KLF15_9bpsite_otteson3a** | **for** | **CACCCCCAA** | **8** | **5.68** |
| 7 | hRho29 | KLF15_9bpsite_otteson3a | for | ACCCCCAAT | 9 | 1.08 |
| 8 | hRho29 | KLF15_9bpsite_otteson3a | for | CTCCCAGAT | 18 | 3.22 |
| 9 | bRho29D1 | KLF15_9bpsite_otteson3a | for | AACGCCCCC | 6 | 1.12 |
| **10** | **bRho29D1** | **KLF15_9bpsite_otteson3a** | **for** | **CGCCCCCAA** | **8** | **6.99** |
| 11 | bRho29D1 | KLF15_9bpsite_otteson3a | for | GCCCCCAAT | 9 | 2.87 |
| 12 | bRho29D1 | KLF15_9bpsite_otteson3a | rev | TCCGAGGTG | 19 | 1.98 |
| 13 | bRho29D4 | KLF15_9bpsite_otteson3a | for | CGCACGCCC | 4 | 2.31 |
| 14 | bRho29D4 | KLF15_9bpsite_otteson3a | for | CACGCCCCC | 6 | 4.83 |
| **15** | **bRho29D4** | **KLF15_9bpsite_otteson3a** | **for** | **CGCCCCCAA** | **8** | **6.99** |
| 16 | bRho29D4 | KLF15_9bpsite_otteson3a | for | GCCCCCAAT | 9 | 2.87 |
| 17 | bRho29D4 | KLF15_9bpsite_otteson3a | rev | TCCGAGGTG | 19 | 1.98 |
| 18 | bRho29D7 | KLF15_9bpsite_otteson3a | for | catccccca | 7 | 2.86 |
| **19** | **bRho29D7** | **KLF15_9bpsite_otteson3a** | **for** | **atcccccaa** | **8** | **3.38** |
| 20 | bRho29D7 | KLF15_9bpsite_otteson3a | for | tcccccaat | 9 | 1.08 |
| 21 | bRho29D7 | KLF15_9bpsite_otteson3a | rev | tccgaggtg | 19 | 1.98 |
| **22** | **bRho29D10** | **KLF15_9bpsite_otteson3a** | **rev** | **tccgaggtg** | **19** | **1.98** |
| **23** | **bRho29D13** | **KLF15_9bpsite_otteson3a** | **for** | **cgcccaaca** | **8** | **4.17** |
| 24 | bRho29D13 | KLF15_9bpsite_otteson3a | rev | tccgaggtg | 19 | 1.98 |
| 25 | bRho29D16 | KLF15_9bpsite_otteson3a | for | AACGCCCCC | 6 | 1.12 |
| **26** | **bRho29D16** | **KLF15_9bpsite_otteson3a** | **for** | **CGCCCCCAC** | **8** | **8.96** |
| 27 | bRho29D16 | KLF15_9bpsite_otteson3a | for | GCCCCCACG | 9 | 1.85 |
| 28 | bRho29D16 | KLF15_9bpsite_otteson3a | for | CCCCCACGA | 10 | 1.13 |
| 29 | bRho29D16 | KLF15_9bpsite_otteson3a | rev | TCCGAGGTG | 19 | 1.98 |
| 30 | bRho29D19 | KLF15_9bpsite_otteson3a | for | AACGCCCCC | 6 | 1.12 |
| **31** | **bRho29D19** | **KLF15_9bpsite_otteson3a** | **for** | **CGCCCCCAA** | **8** | **6.99** |
| 32 | bRho29D19 | KLF15_9bpsite_otteson3a | for | GCCCCCAAT | 9 | 2.87 |
| 33 | bRho29D19 | KLF15_9bpsite_otteson3a | rev | GAAGAGGTG | 19 | 1.79 |
| 34 | bRho29D22 | KLF15_9bpsite_otteson3a | for | AACGCCCCC | 6 | 1.12 |
| **35** | **bRho29D22** | **KLF15_9bpsite_otteson3a** | **for** | **CGCCCCCAA** | **8** | **6.99** |
| 36 | bRho29D22 | KLF15_9bpsite_otteson3a | for | GCCCCCAAT | 9 | 2.87 |
| 37 | bRho29D25 | KLF15_9bpsite_otteson3a | for | AACGCCCCC | 6 | 1.12 |
| **38** | **bRho29D25** | **KLF15_9bpsite_otteson3a** | **for** | **CGCCCCCAA** | **8** | **6.99** |
| 39 | bRho29D25 | KLF15_9bpsite_otteson3a | for | GCCCCCAAT | 9 | 2.87 |
| **40** | **bRho29D11** | **KLF15_9bpsite_otteson3a** | **for** | **cgcacccaa** | **8** | **3.16** |
| 41 | bRho29D11 | KLF15_9bpsite_otteson3a | for | cacccaatc | 10 | 1.79 |
| **42** | **bRho29D7+D13** | **KLF15_9bpsite_otteson3a** | **for** | **catcccaac** | **7** | **3.82** |
| 43 | bRho29D7+D13 | KLF15_9bpsite_otteson3a | rev | tccgaggtg | 19 | 1.98 |
| **44** | **bRho29D8_13** | **KLF15_9bpsite_otteson3a** | **rev** | **tccgaggtg** | **19** | **1.98** |

#### ----------------------------------------------------------------------------------------------------------

#### List of putative binding sites for matrix 4: klf15_6040_9bpmatrix4 (cut-off score 1)

Good competitors CAPS; poor competitors, lowercase.

Highest score for each oligo in bold, underlined

Highest score for competitor: 8.68

Lowest score for compeitor: 6.36

Highest score for non-competitor: 5.05

Difference: 1.31

|  | seq name | TF name | BS dir | BS seq | BS pos | BS score |
| --- | --- | --- | --- | --- | --- | --- |
| **1** | **bRho29** | **klf15_6040_9bpmatrix4** | **for** | **TAACGCCCC** | **5** | **7.34** |
| 2 | bRho29 | klf15_6040_9bpmatrix4 | for | CCAATCTCC | 13 | 2.56 |
| **3** | **hRho29** | **klf15_6040_9bpmatrix4** | **for** | **GAACACCCC** | **5** | **6.36** |
| 4 | hRho29 | klf15_6040_9bpmatrix4 | for | ACACCCCCA | 7 | 2.69 |
| 5 | hRho29 | klf15_6040_9bpmatrix4 | for | CCAATCTCC | 13 | 2.56 |
| 6 | hRho29 | klf15_6040_9bpmatrix4 | for | CAATCTCCC | 14 | 1.55 |
| 7 | hRho29 | klf15_6040_9bpmatrix4 | for | AATCTCCCA | 15 | 1.47 |
| **8** | **bRho29D1** | **klf15_6040_9bpmatrix4** | **for** | **TAACGCCCC** | **5** | **7.34** |
| 9 | bRho29D1 | klf15_6040_9bpmatrix4 | for | CCAATCTCC | 13 | 2.56 |
| **10** | **bRho29D4** | **klf15_6040_9bpmatrix4** | **for** | **GCACGCCCC** | **5** | **8.68** |
| 11 | bRho29D4 | klf15_6040_9bpmatrix4 | for | CACGCCCCC | 6 | 4.30 |
| 12 | bRho29D4 | klf15_6040_9bpmatrix4 | for | CCAATCTCC | 13 | 2.56 |
| 13 | bRho29D7 | klf15_6040_9bpmatrix4 | for | atacatccc | 4 | 1.11 |
| 14 | bRho29D7 | klf15_6040_9bpmatrix4 | for | acatccccc | 6 | 2.81 |
| **15** | **bRho29D7** | **klf15_6040_9bpmatrix4** | **for** | **catccccca** | **7** | **5.05** |
| 16 | bRho29D7 | klf15_6040_9bpmatrix4 | for | ccaatctcc | 13 | 2.56 |
| **17** | **bRho29D10** | **klf15_6040_9bpmatrix4** | **for** | **ccaatctcc** | **13** | **2.56** |
| **18** | **bRho29D13** | **klf15_6040_9bpmatrix4** | **for** | **taacgccca** | **5** | **3.51** |
| 19 | bRho29D13 | klf15_6040_9bpmatrix4 | for | caacatctc | 12 | 2.07 |
| 20 | bRho29D13 | klf15_6040_9bpmatrix4 | for | catctccga | 15 | 1.34 |
| **21** | **bRho29D16** | **klf15_6040_9bpmatrix4** | **for** | **TAACGCCCC** | **5** | **7.34** |
| 22 | bRho29D16 | klf15_6040_9bpmatrix4 | for | CCACGATCC | 13 | 2.81 |
| 23 | bRho29D19 | klf15_6040_9bpmatrix4 | for | TAACGCCCC | 5 | 7.34 |
| **24** | **bRho29D22** | **klf15_6040_9bpmatrix4** | **for** | **TAACGCCCC** | **5** | **7.34** |
| 25 | bRho29D22 | klf15_6040_9bpmatrix4 | for | CCAATCTCC | 13 | 2.56 |
| **26** | **bRho29D25** | **klf15_6040_9bpmatrix4** | **for** | **TAACGCCCC** | **5** | **7.34** |
| 27 | bRho29D25 | klf15_6040_9bpmatrix4 | for | CCAATCTCC | 13 | 2.56 |
| **28** | **bRho29D11** | **klf15_6040_9bpmatrix4** | **for** | **taacgcacc** | **5** | **3.51** |
| 29 | bRho29D11 | klf15_6040_9bpmatrix4 | for | ccaatctcc | 13 | 2.56 |
| 30 | bRho29D7+D13 | klf15_6040_9bpmatrix4 | for | atacatccc | 4 | 1.11 |
| 31 | bRho29D7+D13 | klf15_6040_9bpmatrix4 | for | catcccaac | 7 | 1.34 |
| **32** | **bRho29D7+D13** | **klf15_6040_9bpmatrix4** | **for** | **caacatctc** | **12** | **2.07** |
| 33 | bRho29D7+D13 | klf15_6040_9bpmatrix4 | for | catctccga | 15 | 1.34 |

----------------------------------------------------------------------------------------------------

4. List of top matrices for **KLF15_unknownsite_otteson** binding site

(sorted by information content)

Assume unknown binding site length

Assume 60%AT/40% GC in genome

sequence 1: KR-a_rev

sequence 2: KR-b_rev

sequence 3: KR-c_for

sequence 4: KR-d_for

sequence 5: KR-e_for

sequence 6: KR-f_rev

sequence 7: KIa_rev

sequence 8: KIb_for

sequence 9: KI-c_for

--------------------------------------------------------------------------------

**MATRIX 1**

expected frequency = 6.59661E-16

**alignment matrix**

A 4 0 1 0 0 0 0 3 3 1 0 1

C 4 7 0 9 9 9 9 2 4 8 2 5

G 0 1 5 0 0 0 0 1 1 0 1 1

T 1 1 3 0 0 0 0 3 1 0 6 2

**sequence alignment**

seq position motif

1 12 C C T C C C C C C C C A

2 10 C C T C C C C T C C C T

3 8 A C G C C C C C A A T C

4 4 T G G C C C C A C C T G

5 3 A C G C C C C G C C T T

6 7 C T G C C C C A A C T C

7 10 A C T C C C C T G C G C

8 7 A C A C C C C A A C T C

9 7 C C G C C C C T T C T C

--------------------------------------------------------------------------------

**MATRIX 2**

expected frequency = 1.39564E-15

**alignment matrix**

A 0 0 1 4 1 1 0 0 0 0

C 8 9 3 3 6 3 9 6 8 9

G 1 0 0 0 2 1 0 0 0 0

T 0 0 5 2 0 4 0 3 1 0

**sequence alignment**

seq position motif

1 12 C C T C C C C C C C

2 10 C C T C C C C T C C

3 13 C C C A A T C T C C

4 1 C C T T G G C C C C

5 6 C C C C G C C T T C

6 12 C C A A C T C C C C

7 7 C C C A C T C C C C

8 4 G C T A C A C C C C

9 12 C C T T C T C C C C

-------------------------------------------------------------------------------

**MATRIX 3**

expected frequency = 1.4888E-15

**alignment matrix**

A 3 0 0 0 3 3 2 0 0 0 0

C 1 8 9 6 6 4 4 9 3 7 8

G 5 1 0 0 0 1 0 0 0 1 1

T 0 0 0 3 0 1 3 0 6 1 0

**sequence alignment**

seq position motif

1 11 A C C T C C C C C C C

2 9 A C C T C C C C T C C

3 12 C C C C A A T C T C C

4 6 G C C C C A C C T G G

5 5 G C C C C G C C T T C

6 9 G C C C C A A C T C C

7 6 G C C C A C T C C C C

8 3 A G C T A C A C C C C

9 9 G C C C C T T C T C C

--------------------------------------------------------------------------------

**MATRIX 4**

expected frequency = 2.03769E-15

**alignment matrix**

A 3 4 0 1 0 0 0 0 2 4 1 0 1

C 4 4 7 0 8 9 9 9 3 3 8 3 5

G 0 0 0 5 1 0 0 0 1 1 0 1 0

T 2 1 2 3 0 0 0 0 3 1 0 5 3

**sequence alignment**

seq position motif

1 11 A C C T C C C C C C C C A

2 9 A C C T C C C C T C C C T

3 7 A A C G C C C C C A A T C

4 2 C T T G G C C C C A C C T

5 2 C A C G C C C C G C C T T

6 6 C C T G C C C C A A C T C

7 9 C A C T C C C C T G C G C

8 6 T A C A C C C C A A C T C

9 6 T C C G C C C C T T C T C

#### ------------------------------------------------------------------------------------------------------

**5.** Analysis to determine cutoff score for identification of KLF15 binding sites

Scores for bRho29, hRho29, bRho29-mutations oligonucleotides

#### List of putative binding sites for matrix 1: KLF15_unknownsite_otteson1 (cut-off score 1)

Good competitors CAPS; poor competitors, lowercase.

Highest score for each oligo in bold, underlined

Highest score for competitor: 9.78

Lowest score for compeitor: 7.98

Highest score for non-competitor: 6.95

Difference: 1.03

|  | seq name | TF name | BS dir | BS seq | BS pos | BS score | |
| --- | --- | --- | --- | --- | --- | --- | --- |
| **1** | **bRho29** | **KLF15_unknownsite_otteson1** | **for** | **ACGCCCCCAATC** | **7** | **9.78** | |
| 2 | bRho29 | KLF15_unknownsite_otteson1 | for | CGCCCCCAATCT | 8 | 1.78 | |
| 3 | bRho29 | KLF15_unknownsite_otteson1 | for | GCCCCCAATCTC | 9 | 1.34 | |
| **4** | **hRho29** | **KLF15_unknownsite_otteson1** | **for** | **ACACCCCCAATC** | **7** | **7.98** | |
| 5 | hRho29 | KLF15_unknownsite_otteson1 | for | ACCCCCAATCTC | 9 | 4.00 | |
| **6** | **bRho29D1** | **KLF15_unknownsite_otteson1** | **for** | **ACGCCCCCAATC** | **7** | **9.78** | |
| 7 | bRho29D1 | KLF15_unknownsite_otteson1 | for | CGCCCCCAATCT | 8 | 1.78 | |
| 8 | bRho29D1 | KLF15_unknownsite_otteson1 | for | GCCCCCAATCTC | 9 | 1.34 | |
| 9 | bRho29D4 | KLF15_unknownsite_otteson1 | for | ACGCACGCCCCC | 3 | 4.37 | |
| **10** | **bRho29D4** | **KLF15_unknownsite_otteson1** | **for** | **ACGCCCCCAATC** | **7** | **9.78** |  |
| 11 | bRho29D4 | KLF15_unknownsite_otteson1 | for | CGCCCCCAATCT | 8 | 1.78 | |
| 12 | bRho29D4 | KLF15_unknownsite_otteson1 | for | GCCCCCAATCTC | 9 | 1.34 | |
| **13** | **bRho29D7** | **KLF15_unknownsite_otteson1** | **for** | **catcccccaatc** | **7** | **5.72** | |
| 14 | bRho29D7 | KLF15_unknownsite_otteson1 | for | atcccccaatct | 8 | 1.07 | |
| 15 | bRho29D7 | KLF15_unknownsite_otteson1 | for | tcccccaatctc | 9 | 2.80 | |
| **16** | **bRho29D13** | **KLF15_unknownsite_otteson1** | **for** | **acgcccaacatc** | **7** | **6.59** | |
| **17** | **bRho29D16** | **KLF15_unknownsite_otteson1** | **for** | **ACGCCCCCACGA** | **7** | **8.98** | |
| **18** | **bRho29D19** | **KLF15_unknownsite_otteson1** | **for** | **ACGCCCCCAATC** | **7** | **9.78** | |
| 19 | bRho29D19 | KLF15_unknownsite_otteson1 | for | CGCCCCCAATCG | 8 | 1.54 | |
| **20** | **bRho29D22** | **KLF15_unknownsite_otteson1** | **for** | **ACGCCCCCAATC** | **7** | **9.78** | |
| 21 | bRho29D22 | KLF15_unknownsite_otteson1 | for | CGCCCCCAATCT | 8 | 1.78 | |
| 22 | bRho29D22 | KLF15_unknownsite_otteson1 | for | GCCCCCAATCTC | 9 | 1.34 | |
| **23** | **bRho29D25** | **KLF15_unknownsite_otteson1** | **for** | **ACGCCCCCAATC** | **7** | **9.78** | |
| 24 | bRho29D25 | KLF15_unknownsite_otteson1 | for | CGCCCCCAATCT | 8 | 1.78 | |
| 25 | bRho29D25 | KLF15_unknownsite_otteson1 | for | GCCCCCAATCTC | 9 | 1.34 | |
| **26** | **bRho29D11** | **KLF15_unknownsite_otteson1** | **for** | **acgcacccaatc** | **7** | **5.95** | |
| 27 | bRho29D11 | KLF15_unknownsite_otteson1 | for | gcacccaatctc | 9 | 2.80 | |
| 28 | bRho29D7+D13 | KLF15_unknownsite_otteson1 | for | acatcccaacat | 6 | 2.13 | |
| **29** | **bRho29D7+D13** | **KLF15_unknownsite_otteson1** | **for** | **catcccaacatc** | **7** | **2.53** | |
| **30** | **bRho29D8_13** | **KLF15_unknownsite_otteson1** | **for** | **aagcccacaatc** | **7** | **2.37** | |

-------------------------------------------------------------------------------------------------------

List of putative binding sites for matrix 2: **KLF15_unknownsite_otteson2** (cut-off score 1)

Good competitors CAPS; poor competitors, lowercase.

Highest score for each oligo in bold, underlined

Highest score for competitor: 7.86

Lowest score for compeitor: 4.46

Highest score for non-competitor: 7.86

Difference: 0.0

|  | seq name | TF name | BS dir | BS seq | BS pos | BS score |
| --- | --- | --- | --- | --- | --- | --- |
| 1 | bRho29 | KLF15_unknownsite_otteson2 | for | ATAACGCCCC | 4 | 1.14 |
| 2 | bRho29 | KLF15_unknownsite_otteson2 | for | TAACGCCCCC | 5 | 1.20 |
| 3 | bRho29 | KLF15_unknownsite_otteson2 | for | CCCCCAATCT | 10 | 1.08 |
| 4 | bRho29 | KLF15_unknownsite_otteson2 | for | CCCCAATCTC | 11 | 1.72 |
| **5** | **bRho29** | **KLF15_unknownsite_otteson2** | **for** | **CCCAATCTCC** | **12** | **7.86** |
| 6 | hRho29 | KLF15_unknownsite_otteson2 | for | GAACACCCCC | 5 | 2.05 |
| 7 | hRho29 | KLF15_unknownsite_otteson2 | for | CCCCCAATCT | 10 | 1.08 |
| 8 | hRho29 | KLF15_unknownsite_otteson2 | for | CCCCAATCTC | 11 | 1.72 |
| **9** | **hRho29** | **KLF15_unknownsite_otteson2** | **for** | **CCCAATCTCC** | **12** | **7.86** |
| 10 | hRho29 | KLF15_unknownsite_otteson2 | for | CCAATCTCCC | 13 | 2.40 |
| 11 | hRho29 | KLF15_unknownsite_otteson2 | for | CAATCTCCCA | 14 | 1.26 |
| 12 | bRho29D1 | KLF15_unknownsite_otteson2 | for | CATAACGCCC | 3 | 1.44 |
| 13 | bRho29D1 | KLF15_unknownsite_otteson2 | for | ATAACGCCCC | 4 | 1.14 |
| 14 | bRho29D1 | KLF15_unknownsite_otteson2 | for | TAACGCCCCC | 5 | 1.20 |
| 15 | bRho29D1 | KLF15_unknownsite_otteson2 | for | CCCCCAATCT | 10 | 1.08 |
| 16 | bRho29D1 | KLF15_unknownsite_otteson2 | for | CCCCAATCTC | 11 | 1.72 |
| **17** | **bRho29D1** | **KLF15_unknownsite_otteson2** | **for** | **CCCAATCTCC** | **12** | **7.86** |
| 18 | bRho29D4 | KLF15_unknownsite_otteson2 | for | CGCACGCCCC | 4 | 6.16 |
| 19 | bRho29D4 | KLF15_unknownsite_otteson2 | for | GCACGCCCCC | 5 | 6.82 |
| 20 | bRho29D4 | KLF15_unknownsite_otteson2 | for | CCCCCAATCT | 10 | 1.08 |
| 21 | bRho29D4 | KLF15_unknownsite_otteson2 | for | CCCCAATCTC | 11 | 1.72 |
| **22** | **bRho29D4** | **KLF15_unknownsite_otteson2** | **for** | **CCCAATCTCC** | **12** | **7.86** |
| 23 | bRho29D7 | KLF15_unknownsite_otteson2 | for | acatccccca | 6 | 1.49 |
| 24 | bRho29D7 | KLF15_unknownsite_otteson2 | for | cccccaatct | 10 | 1.08 |
| 25 | bRho29D7 | KLF15_unknownsite_otteson2 | for | ccccaatctc | 11 | 1.72 |
| **26** | **bRho29D7** | **KLF15_unknownsite_otteson2** | **for** | **cccaatctcc** | **12** | **7.86** |
| **27** | **bRho29D10** | **KLF15_unknownsite_otteson2** | **for** | **accaatctcc** | **12** | **4.15** |
| 28 | bRho29D13 | KLF15_unknownsite_otteson2 | for | ccaacatctc | 11 | 2.27 |
| **29** | **bRho29D13** | **KLF15_unknownsite_otteson2** | **for** | **caacatctcc** | **12** | **2.83** |
| 30 | bRho29D16 | KLF15_unknownsite_otteson2 | for | ATAACGCCCC | 4 | 1.14 |
| 31 | bRho29D16 | KLF15_unknownsite_otteson2 | for | TAACGCCCCC | 5 | 1.20 |
| **32** | **bRho29D16** | **KLF15_unknownsite_otteson2** | **for** | **CCCACGATCC** | **12** | **5.13** |
| 33 | bRho29D19 | KLF15_unknownsite_otteson2 | for | ATAACGCCCC | 4 | 1.14 |
| 34 | bRho29D19 | KLF15_unknownsite_otteson2 | for | TAACGCCCCC | 5 | 1.20 |
| 35 | bRho29D19 | KLF15_unknownsite_otteson2 | for | CCCCCAATCG | 10 | 1.08 |
| **36** | **bRho29D19** | **KLF15_unknownsite_otteson2** | **rev** | **GAAGAGGTGC** | **19** | **4.46** |
| 37 | bRho29D22 | KLF15_unknownsite_otteson2 | for | ATAACGCCCC | 4 | 1.14 |
| 38 | bRho29D22 | KLF15_unknownsite_otteson2 | for | TAACGCCCCC | 5 | 1.20 |
| 39 | bRho29D22 | KLF15_unknownsite_otteson2 | for | CCCCCAATCT | 10 | 1.08 |
| 40 | bRho29D22 | KLF15_unknownsite_otteson2 | for | CCCCAATCTC | 11 | 1.72 |
| **41** | **bRho29D22** | **KLF15_unknownsite_otteson2** | **for** | **CCCAATCTCC** | **12** | **7.86** |
| 42 | bRho29D25 | KLF15_unknownsite_otteson2 | for | ATAACGCCCC | 4 | 1.14 |
| 43 | bRho29D25 | KLF15_unknownsite_otteson2 | for | TAACGCCCCC | 5 | 1.20 |
| 44 | bRho29D25 | KLF15_unknownsite_otteson2 | for | CCCCCAATCT | 10 | 1.08 |
| 45 | bRho29D25 | KLF15_unknownsite_otteson2 | for | CCCCAATCTC | 11 | 1.72 |
| **46** | **bRho29D25** | **KLF15_unknownsite_otteson2** | **for** | **CCCAATCTCC** | **12** | **7.86** |
| **47** | **bRho29D11** | **KLF15_unknownsite_otteson2** | **for** | **cccaatctcc** | **12** | **7.86** |
| 48 | bRho29D7+D13 | KLF15_unknownsite_otteson2 | for | ccaacatctc | 11 | 2.27 |
| **49** | **bRho29D7+D13** | **KLF15_unknownsite_otteson2** | **for** | **caacatctcc** | **12** | **2.83** |
| **50** | **bRho29D8_13** | **KLF15_unknownsite_otteson2** | **for** | **cacaatctcc** | **12** | **4.03** |

#### ---------------------------------------------------------------------------------------------------------

#### List of putative binding sites for matrix 3: KLF15_unknownsite_otteson3 (cut-off score 1)

Good competitors CAPS; poor competitors, lowercase.

Highest score for each oligo in bold, underlined

Highest score for competitor: 8.82

Lowest score for compeitor: 3.23

Highest score for non-competitor: 9.42

Difference: -6.19

|  | seq name | TF name | BS dir | BS seq | BS pos | BS score |
| --- | --- | --- | --- | --- | --- | --- |
| 1 | bRho29 | KLF15_unknownsite_otteson3 | for | GCCCCCAATCT | 9 | 4.05 |
| 2 | bRho29 | KLF15_unknownsite_otteson3 | for | CCCCCAATCTC | 10 | 3.26 |
| **3** | **bRho29** | **KLF15_unknownsite_otteson3** | **for** | **CCCCAATCTCC** | **11** | **8.82** |
| 4 | hRho29 | KLF15_unknownsite_otteson3 | for | ACCCCCAATCT | 9 | 3.19 |
| 5 | hRho29 | KLF15_unknownsite_otteson3 | for | CCCCCAATCTC | 10 | 3.26 |
| **6** | **hRho29** | **KLF15_unknownsite_otteson3** | **for** | **CCCCAATCTCC** | **11** | **8.82** |
| 7 | bRho29D1 | KLF15_unknownsite_otteson3 | for | GCATAACGCCC | 2 | 1.97 |
| 8 | bRho29D1 | KLF15_unknownsite_otteson3 | for | GCCCCCAATCT | 9 | 4.05 |
| 9 | bRho29D1 | KLF15_unknownsite_otteson3 | for | CCCCCAATCTC | 10 | 3.26 |
| **10** | **bRho29D1** | **KLF15_unknownsite_otteson3** | **for** | **CCCCAATCTCC** | **11** | **8.82** |
| 11 | bRho29D4 | KLF15_unknownsite_otteson3 | for | ACGCACGCCCC | 3 | 3.57 |
| 12 | bRho29D4 | KLF15_unknownsite_otteson3 | for | CGCACGCCCCC | 4 | 4.26 |
| 13 | bRho29D4 | KLF15_unknownsite_otteson3 | for | GCACGCCCCCA | 5 | 1.36 |
| 14 | bRho29D4 | KLF15_unknownsite_otteson3 | for | GCCCCCAATCT | 9 | 4.05 |
| 15 | bRho29D4 | KLF15_unknownsite_otteson3 | for | CCCCCAATCTC | 10 | 3.26 |
| **16** | **bRho29D4** | **KLF15_unknownsite_otteson3** | **for** | **CCCCAATCTCC** | **11** | **8.82** |
| 17 | bRho29D7 | KLF15_unknownsite_otteson3 | for | atacatccccc | 4 | 1.32 |
| 18 | bRho29D7 | KLF15_unknownsite_otteson3 | for | cccccaatctc | 10 | 3.26 |
| **19** | **bRho29D7** | **KLF15_unknownsite_otteson3** | **for** | **ccccaatctcc** | **11** | **8.82** |
| **20** | **bRho29D10** | **KLF15_unknownsite_otteson3** | **for** | **aaccaatctcc** | **11** | **5.72** |
| 21 | bRho29D13 | KLF15_unknownsite_otteson3 | for | cgcccaacatc | 8 | 2.40 |
| **22** | **bRho29D13** | **KLF15_unknownsite_otteson3** | **for** | **gcccaacatct** | **9** | **3.39** |
| 23 | bRho29D13 | KLF15_unknownsite_otteson3 | for | ccaacatctcc | 11 | 2.59 |
| 24 | bRho29D16 | KLF15_unknownsite_otteson3 | for | ACGCCCCCACG | 7 | 2.95 |
| 25 | bRho29D16 | KLF15_unknownsite_otteson3 | for | GCCCCCACGAT | 9 | 1.26 |
| 26 | bRho29D16 | KLF15_unknownsite_otteson3 | for | CCCCCACGATC | 10 | 1.50 |
| **27** | **bRho29D16** | **KLF15_unknownsite_otteson3** | **for** | **CCCCACGATCC** | **11** | **3.23** |
| **28** | **bRho29D19** | **KLF15_unknownsite_otteson3** | **for** | **GCCCCCAATCG** | **9** | **5.84** |
| 29 | bRho29D19 | KLF15_unknownsite_otteson3 | rev | CGAAGAGGTGC | 18 | 1.44 |
| 30 | bRho29D22 | KLF15_unknownsite_otteson3 | for | GCCCCCAATCT | 9 | 4.05 |
| 31 | bRho29D22 | KLF15_unknownsite_otteson3 | for | CCCCCAATCTC | 10 | 3.26 |
| **32** | **bRho29D22** | **KLF15_unknownsite_otteson3** | **for** | **CCCCAATCTCC** | **11** | **8.82** |
| 33 | bRho29D25 | KLF15_unknownsite_otteson3 | for | GCCCCCAATCT | 9 | 4.05 |
| 34 | bRho29D25 | KLF15_unknownsite_otteson3 | for | CCCCCAATCTC | 10 | 3.26 |
| **35** | **bRho29D25** | **KLF15_unknownsite_otteson3** | **for** | **CCCCAATCTCC** | **11** | **8.82** |
| **36** | **bRho29D11** | **KLF15_unknownsite_otteson3** | **for** | **acccaatctcc** | **11** | **9.43** |
| 37 | bRho29D7+D13 | KLF15_unknownsite_otteson3 | for | atcccaacatc | 8 | 1.22 |
| **38** | **bRho29D7+D13** | **KLF15_unknownsite_otteson3** | **for** | **ccaacatctcc** | **11** | **2.59** |
| 39 | bRho29D8_13 | KLF15_unknownsite_otteson3 | for | gcccacaatct | 9 | 3.02 |
| **40** | **bRho29D8_13** | **KLF15_unknownsite_otteson3** | **for** | **ccacaatctcc** | **11** | **4.99** |
| **41** | **IRBP1** | **KLF15_unknownsite_otteson3** | **rev** | **ggattaaaggc** | **6** | **1.72** |

#### --------------------------------------------------------------------------------------------------------

#### List of putative binding sites for matrix 4: KLF15_unknownsite_otteson4 (cut-off score 1)

Good competitors CAPS; poor competitors, lowercase.

Highest score for each oligo in bold, underlined

Highest score for competitor: 10.22

Lowest score for compeitor: 9.95

Highest score for non-competitor: 6.39

Difference: 3.20

|  | seq name | TF name | BS dir | BS seq | BS pos | BS score |
| --- | --- | --- | --- | --- | --- | --- |
| **1** | **bRho29** | **KLF15_unknownsite_otteson4** | **for** | **AACGCCCCCAATC** | **6** | **10.22** |
| 2 | bRho29 | KLF15_unknownsite_otteson4 | for | CGCCCCCAATCTC | 8 | 1.42 |
| 3 | hRho29 | KLF15_unknownsite_otteson4 | for | AACACCCCCAATC | 6 | 8.42 |
| 4 | hRho29 | KLF15_unknownsite_otteson4 | for | CACCCCCAATCTC | 8 | 4.08 |
| **5** | **bRho29D1** | **KLF15_unknownsite_otteson4** | **for** | **AACGCCCCCAATC** | **6** | **10.22** |
| 6 | bRho29D1 | KLF15_unknownsite_otteson4 | for | CGCCCCCAATCTC | 8 | 1.42 |
| 7 | bRho29D4 | KLF15_unknownsite_otteson4 | for | TACGCACGCCCCC | 2 | 4.45 |
| **8** | **bRho29D4** | **KLF15_unknownsite_otteson4** | **for** | **CACGCCCCCAATC** | **6** | **10.86** |
| 9 | bRho29D4 | KLF15_unknownsite_otteson4 | for | CGCCCCCAATCTC | 8 | 1.42 |
| **10** | **bRho29D7** | **KLF15_unknownsite_otteson4** | **for** | **acatcccccaatc** | **6** | **6.16** |
| 11 | bRho29D7 | KLF15_unknownsite_otteson4 | for | catcccccaatct | 7 | 2.89 |
| 12 | bRho29D7 | KLF15_unknownsite_otteson4 | for | atcccccaatctc | 8 | 2.24 |
| **13** | **bRho29D13** | **KLF15_unknownsite_otteson4** | **for** | **aacgcccaacatc** | **6** | **5.76** |
| **14** | **bRho29D16** | **KLF15_unknownsite_otteson4** | **for** | **AACGCCCCCACGA** | **6** | **9.59** |
| **15** | **bRho29D19** | **KLF15_unknownsite_otteson4** | **for** | **AACGCCCCCAATC** | **6** | **10.22** |
| **16** | **bRho29D22** | **KLF15_unknownsite_otteson4** | **for** | **AACGCCCCCAATC** | **6** | **10.22** |
| 17 | bRho29D22 | KLF15_unknownsite_otteson4 | for | CGCCCCCAATCTC | 8 | 1.42 |
| **18** | **bRho29D25** | **KLF15_unknownsite_otteson4** | **for** | **AACGCCCCCAATC** | **6** | **10.22** |
| 19 | bRho29D25 | KLF15_unknownsite_otteson4 | for | CGCCCCCAATCTC | 8 | 1.42 |
| **20** | **bRho29D11** | **KLF15_unknownsite_otteson4** | **for** | **aacgcacccaatc** | **6** | **6.39** |
| 21 | bRho29D11 | KLF15_unknownsite_otteson4 | for | cgcacccaatctc | 8 | 2.88 |
| **22** | **bRho29D7+D13** | **KLF15_unknownsite_otteson4** | **for** | **tacatcccaacat** | **5** | **2.12** |
| 23 | bRho29D7+D13 | KLF15_unknownsite_otteson4 | for | acatcccaacatc | 6 | 1.70 |
| **24** | **bRho29D8_13** | **KLF15_unknownsite_otteson4** | **for** | **aaagcccacaatc** | **6** | **2.81** |
| **25** | **IRBP2** | **KLF15_unknownsite_otteson4** | **rev** | **tgtcagggccttt** | **6** | **1.58** |

#### ------------------------------------------------------------------------------------------------------------

6. Identification of potential KLF15 binding sites in rhodopsin and IRBP promoters

sequences analyzed:

>bovineRho

aggggtcccaatgccaggccagggcccatcagctgagatgccagagggggacaggggagttgggggcaggcctctgctctttcccagggtccccagcacgccccgccttctccccgaccacagccttctacctggagtcaccttggccccacctggaagccaattaagcccctcgttgcagcagtgaggattaatatgattaataacgcccccaatctccgaggtgctgattcagccgggagcttagggaggggaggtcacttcataagggcctggggggggagttggagccacgagtcgtccagccggagccccgtgtggctgag

>humanRho

tggtccctatttcaaacccaggccaccagactgagctgggaccttgggacagacaagtcatgcagaagttaggggaccttctcctcccttttcctggatcctgagtacctctcctccctgacctcaggcttcctcctagtgtcaccttggcccctcttagaagccaattaggccctcagtttctgcagcggggattaatatgattatgaacacccccaatctcccagatgctgattcagccaggagcttaggagggggaggtcactttataagggtctgggggggtcagaacccagagtcatccagctggagccctgagtggctgag

>chimpRho

ggaccccagactgagctgggaccttgggacagagaagtcatgcagaagttgggggaccttctcctcccttttcctggatcctgagtacctctcctccctgacctcaggcttcctcctagtgtcaccttggcccctcttggaagccaattaggccctcagtttctgcagcggggattaatatgattatgaacacccccaatctcccagatgctgattcagccaggagcttaggagggggaggtcactttataagggtctgggggggtcagaacccagagtcatccagctggagccctgagtggctgagctcaggcttcgcagcattcttgggtg

>dogRho

ggccaccagactgatctgggcagagattcctggggaccagaagagttgggggcggccttctgcccttcccagggtccccagcacctcccctctctggccccaggcttctgtctagagtcaccttggccactcttggaagccaattaggcccctagttgcggcagtggggattattatgattaataacgcccccaatctcccaggtgctgattcagccaggagcttagggaggggaggtcactttataacggcctggggggggggtcagagcctgaagtcgtcctgccggagccctgggtggccaagctcaggcctcagcagcactcttaggac

>mouseRho

aaggttccagtcgcagcctgaggccaccagactgacatggggaggaattcccagaggactctggggcagacaagatgagacaccctttcctttctttacctaagggcctccacccgatgtcaccttggcccctctgcaagccaattaggccccggtggcagcagtgggattagcgttagtatgatatctcgcggatgctgaatcagcctctggcttagggagagaaggtcactttataagggtctggggggggtcagtgcctggagttgcgctgtgggagccgtcagtggctgag

>ratRho

aaagactccagtcacagcctgaggccaccagagtgacatggggagaaattcctgggggactccagggcaaacaagatgagacaccctttctttacctaagggcttccacctgatgtcaccttggcccctctgcaagctaattaggccccagtggcagcagtgggattagtattagtgtgatatctcccggatgctgaatcagcctctggcttagagagagaaggtcactttataagggtctggggggggtcagtgcctggagttgtgctgtgggagccgtaggtagctgag

>bovineIRBP

tcatgtcattctaccagaggtggtgcagccagatgagaccccaacataccttctgggtcaagccacttccgccccttctccccagtccccagggcttatgagcttgaattaaataggattaaaggcttatcagggctgggagctacaccccaactcctgagtttagccccagaccttctgtccgccagcttgagaaggacgagcgagaaggcagctgcgcaggggagtgggctgttgccttcg

>humanIRBP

taatgttatgatatgcagacacaacacagcaagataagatgcaatgtaccttctgggtcaaaccaccctggccactcctccccgatacccagggttgatgtgcttgaattagacaggattaaaggcttactggagctggaagccttgccccaactcaggagtttagccccagaccttctgtccaccagctgagaaggacaagggcggaaggcagctgcacagagcagggccacggccttgc

>chimpIRBP

atgttatgatatgcagacacaacaagcaagataagatgcaatgtaccttctgggtcaaaccaccctggccactcctccccgatacccagggttgatgtgcttgaattagactggattaaaggcttactggagctggaagccttgccccaactcaggagtttagccccagaccttctgtccaccagctgagaaggacaagggtggaaggcagctgcacagagcagggccacggccttgcacacagtccag

>dogIRBP

gtggccgcgcaccgaggtggcacagctcggtgagatccacgcgccttctgggtcaaaccgccgcggcccctgctccccggtccccggggctgacgcgcttgaattagacgggattaaaggcttactggagctgcaagccgcaccccaactcccgagtttagccccagaccttctgtccacccgctgagaaggacgagggaggaaggcggccacacgggcagcgccgtggccttgcgcacagtcaag

>mouseIRBP

ttgtgttattctacacagacatggctcagcaagatgagatgcaatgttccttccgggtcaaaccaccctggcctcttcttcagagtccagctcatgtgcttgaattagacaggattaaaggcttagcagagctggaagcctcacatctaactcccacattgagccccagaccttctgtctgcctgctaagaagggcaagggagagaggcagctgcaggaccacagccttgtacacggagc

>ratIRBP

ttgtgtaattctatgcagacacatctcggcaagatgagatgtgatgttccttccgggtcaaactaccctggcctcttcttctgagtctcctggttcatgcatgcgcttgaattagacaggattaaaggcttaccagagctggaagcctcacgtctaactcccacgttgagccccagaccttctgtctgcctgctaagaagggcaacagagaaaggcagctgcaggagcagggccacagtcttgtacagtcaag

#### List of putative binding sites for klf15_60_40_9bpmatrix2 (cut-off score 4.87)

|  | seq name | chromo some | TF name | BS dir | BS seq | BS pos | BS score |
| --- | --- | --- | --- | --- | --- | --- | --- |
| 1 | bovineRho |  | klf15_60_40_9bpmatrix2 | rev | *gagggggac* | 44 | 6.51 |
| 2 | bovineRho |  | klf15_60_40_9bpmatrix2 | rev | *acaggggag* | 51 | 7.51 |
| 3 | bovineRho |  | klf15_60_40_9bpmatrix2 | for | *gtccccagc* | 89 | 7.08 |
| 4 | bovineRho |  | klf15_60_40_9bpmatrix2 | for | *cgccccgcc* | 99 | 9.78 |
| 5 | bovineRho |  | klf15_60_40_9bpmatrix2 | for | *ctccccgac* | 110 | 8.39 |
| 6 | bovineRho |  | klf15_60_40_9bpmatrix2 | for | *ggccccacc* | 145 | 8.10 |
| 7 | bovineRho |  | klf15_60_40_9bpmatrix2 | for | *cgcccccaa* | 207 | 7.26 |
| 8 | bovineRho |  | klf15_60_40_9bpmatrix2 | rev | *ggaggggag* | 248 | 10.27 |
| 9 | bovineRho |  | klf15_60_40_9bpmatrix2 | rev | *tgggggggg* | 274 | 5.23 |
| 10 | bovineRho |  | klf15_60_40_9bpmatrix2 | rev | *gggggggag* | 276 | 10.01 |
| 11 | humanRho |  | klf15_60_40_9bpmatrix2 | rev | *ttaggggac* | 69 | 5.22 |
| 12 | humanRho |  | klf15_60_40_9bpmatrix2 | for | *ctctcctcc* | 109 | 6.44 |
| 13 | humanRho |  | klf15_60_40_9bpmatrix2 | for | *ggcccctct* | 149 | 6.61 |
| 14 | humanRho |  | klf15_60_40_9bpmatrix2 | for | *cacccccaa* | 211 | 5.68 |
| 15 | humanRho |  | klf15_60_40_9bpmatrix2 | rev | *gagggggag* | 252 | 8.43 |
| 16 | humanRho |  | klf15_60_40_9bpmatrix2 | rev | *gggggggtc* | 279 | 6.89 |
| 17 | chimpRho |  | klf15_60_40_9bpmatrix2 | rev | *ttgggggac* | 49 | 4.96 |
| 18 | chimpRho |  | klf15_60_40_9bpmatrix2 | for | *ctctcctcc* | 89 | 6.44 |
| 19 | chimpRho |  | klf15_60_40_9bpmatrix2 | for | *ggcccctct* | 129 | 6.61 |
| 20 | chimpRho |  | klf15_60_40_9bpmatrix2 | for | *cacccccaa* | 191 | 5.68 |
| 21 | chimpRho |  | klf15_60_40_9bpmatrix2 | rev | *gagggggag* | 232 | 8.43 |
| 22 | chimpRho |  | klf15_60_40_9bpmatrix2 | rev | *gggggggtc* | 259 | 6.89 |
| 23 | dogRho |  | klf15_60_40_9bpmatrix2 | rev | *ttgggggcg* | 46 | 7.26 |
| 24 | dogRho |  | klf15_60_40_9bpmatrix2 | for | *gtccccagc* | 74 | 7.08 |
| 25 | dogRho |  | klf15_60_40_9bpmatrix2 | for | *cacctcccc* | 82 | 4.98 |
| 26 | dogRho |  | klf15_60_40_9bpmatrix2 | for | *ctcccctct* | 85 | 8.15 |
| 27 | dogRho |  | klf15_60_40_9bpmatrix2 | for | *cgcccccaa* | 187 | 7.26 |
| 28 | dogRho |  | klf15_60_40_9bpmatrix2 | rev | *ggaggggag* | 228 | 10.27 |
| 29 | dogRho |  | klf15_60_40_9bpmatrix2 | rev | *tgggggggg* | 254 | 5.23 |
| 30 | dogRho |  | klf15_60_40_9bpmatrix2 | rev | *ggggggggg* | 255 | 7.35 |
| 31 | dogRho |  | klf15_60_40_9bpmatrix2 | rev | *ggggggggg* | 256 | 7.35 |
| 32 | dogRho |  | klf15_60_40_9bpmatrix2 | rev | *gggggggtc* | 258 | 6.89 |
| 33 | mouseRho |  | klf15_60_40_9bpmatrix2 | for | *ggcccctct* | 127 | 6.61 |
| 34 | mouseRho |  | klf15_60_40_9bpmatrix2 | for | *ggccccggt* | 148 | 5.10 |
| 35 | mouseRho |  | klf15_60_40_9bpmatrix2 | rev | *tgggggggg* | 245 | 5.23 |
| 36 | mouseRho |  | klf15_60_40_9bpmatrix2 | rev | *gggggggtc* | 247 | 6.89 |
| 37 | ratRho |  | klf15_60_40_9bpmatrix2 | for | *ggcccctct* | 123 | 6.61 |
| 38 | ratRho |  | klf15_60_40_9bpmatrix2 | for | *ggccccagt* | 144 | 5.34 |
| 39 | ratRho |  | klf15_60_40_9bpmatrix2 | rev | *tgggggggg* | 241 | 5.23 |
| 40 | ratRho |  | klf15_60_40_9bpmatrix2 | rev | *gggggggtc* | 243 | 6.89 |
| 41 | bovineIRBP |  | klf15_60_40_9bpmatrix2 | for | *gaccccaac* | 37 | 5.51 |
| 42 | bovineIRBP |  | klf15_60_40_9bpmatrix2 | for | *cgccccttc* | 70 | 9.07 |
| 43 | bovineIRBP |  | klf15_60_40_9bpmatrix2 | for | *ctccccagt* | 78 | 6.88 |
| 44 | bovineIRBP |  | klf15_60_40_9bpmatrix2 | rev | *ggctgggag* | 134 | 5.57 |
| 45 | bovineIRBP |  | klf15_60_40_9bpmatrix2 | for | *caccccaac* | 146 | 7.43 |
| 46 | bovineIRBP |  | klf15_60_40_9bpmatrix2 | rev | *gcaggggag* | 219 | 9.63 |
| 47 | humanIRBP |  | klf15_60_40_9bpmatrix2 | for | *ggccactcc* | 70 | 4.90 |
| 48 | humanIRBP |  | klf15_60_40_9bpmatrix2 | for | *cactcctcc* | 73 | 5.24 |
| 49 | humanIRBP |  | klf15_60_40_9bpmatrix2 | for | *ctcctcccc* | 75 | 6.18 |
| 50 | humanIRBP |  | klf15_60_40_9bpmatrix2 | for | *ctccccgat* | 78 | 6.27 |
| 51 | humanIRBP |  | klf15_60_40_9bpmatrix2 | for | *tgccccaac* | 146 | 5.30 |
| 52 | chimpIRBP |  | klf15_60_40_9bpmatrix2 | for | *ggccactcc* | 67 | 4.90 |
| 53 | chimpIRBP |  | klf15_60_40_9bpmatrix2 | for | *cactcctcc* | 70 | 5.24 |
| 54 | chimpIRBP |  | klf15_60_40_9bpmatrix2 | for | *ctcctcccc* | 72 | 6.18 |
| 55 | chimpIRBP |  | klf15_60_40_9bpmatrix2 | for | *ctccccgat* | 75 | 6.27 |
| 56 | chimpIRBP |  | klf15_60_40_9bpmatrix2 | for | *tgccccaac* | 143 | 5.30 |
| 57 | dogIRBP |  | klf15_60_40_9bpmatrix2 | for | *cgcgccttc* | 40 | 5.24 |
| 58 | dogIRBP |  | klf15_60_40_9bpmatrix2 | for | *cgccgcggc* | 59 | 5.31 |
| 59 | dogIRBP |  | klf15_60_40_9bpmatrix2 | for | *ggcccctgc* | 65 | 8.09 |
| 60 | dogIRBP |  | klf15_60_40_9bpmatrix2 | for | *ctccccggt* | 73 | 6.64 |
| 61 | dogIRBP |  | klf15_60_40_9bpmatrix2 | for | *caccccaac* | 141 | 7.43 |
| 62 | mouseIRBP |  | klf15_60_40_9bpmatrix2 | rev | *gcaagggag* | 195 | 5.80 |
